# Early-life prophylactic antibiotic treatment disturbs the stability of the gut microbiota and increases susceptibility to H9N2 AIV in chicks

**DOI:** 10.1186/s40168-023-01609-8

**Published:** 2023-07-26

**Authors:** Xianghui Liang, Zhipeng Zhang, Hai Wang, Xingbang Lu, Wen Li, Haoran Lu, Ayan Roy, Xuejuan Shen, David M. Irwin, Yongyi Shen

**Affiliations:** 1 Guangdong Laboratory for Lingnan Modern Agriculture, State Key Laboratory for Animal Disease Control and Prevention, Center for Emerging and Zoonotic Diseases, College of Veterinary Medicine, South China Agricultural University, Guangzhou, 510642 China; 2 School of Mathematics, Sun Yat-Sen University, Guangzhou, 510275 China; 3 Mailman School of Public Health, Columbia University, New York, 10032 USA; 4 Department of Laboratory Medicine and Pathobiology, University of Toronto, Toronto, M5S1A8 Canada; 5 Banting and Best Diabetes Centre, University of Toronto, Toronto, M5S1A8 Canada; 6 Guangdong Provincial Key Laboratory of Zoonosis Prevention and Control, Guangzhou, 510642 China

**Keywords:** Prophylactic antibiotic, Chicken, Gut microbiota, H9N2 influenza virus, Disease resistance, Antibiotic resistance genes

## Abstract

**Background:**

Antibiotics are widely used for prophylactic therapy and for improving the growth performance of chicken. The problem of bacterial drug resistance caused by antibiotic abuse has previously attracted extensive attention; however, the influence of early-day use of prophylactic antibiotics on the gut microflora and on the disease resistance ability in chicks has not been explored. Here, we comprehensively evaluate the growth performance, gut microbial dynamics, level of antibiotic resistance genes (ARGs) in the gut microbial community, and resistance to H9N2 avian influenza virus (AIV) in chickens following long-term and short-term early-day prophylactic antibiotic treatment.

**Results:**

Unexpectedly, long-term prophylactic enrofloxacin treatment slowed the growth rate of chickens, whereas short-term antibiotics treatments were found to increase the growth rate, but these changes were not statistically significant. Strikingly, expansions of *Escherichia-Shigella* populations were observed in early-life prophylactic antibiotics-treated groups of chickens, which is in contrast to the general perception that antibiotics should control their pathogenicity in chicks. The gut microbiota composition of chickens treated long term with antibiotics or received early-day antibiotics treatment tend to be more dramatically disturbed compared to the gut microbiome of chickens treated with antibiotics for a short term at a later date, especially after H9N2 AIV infection.

**Conclusions:**

Our data provide evidence that early-day and long-term antibiotic treatments have a more adverse effect on the intestinal microbiome of chickens, compared to short-term late age antibiotic treatment. Furthermore, our metagenomic data reveal that both long-term and short-term antibiotic treatment increase the relative abundance of ARGs. Our findings highlight the adverse effects of prophylactic antibiotic treatment and provide a theoretical basis for the cautious administration of antibiotics in food-producing animal management.

Video Abstract

**Supplementary Information:**

The online version contains supplementary material available at 10.1186/s40168-023-01609-8.

## Background

Antibiotics have been widely used over the past 50 years to improve growth performance and control bacterial diseases in agricultural animals [1]. Therapeutic antibiotics are typically used for a short period of time to treat sick animals after a disease outbreak [1]. In contrast, prophylactic antibiotics, commonly at a low dose, are persistently administered to animals without disease symptoms for long periods of time to prevent diseases in animals. For instance, beef cattle in North America frequently receive veterinary antibiotics to control bovine respiratory disease when and after they are transported long-distances [2]. Although *Escherichia coli* is part of the normal flora of poultry, it can also act as an opportunistic agent causing colibacillosis, which is a leading causes of mortality (especially in young chicks) and results in decreased meat and egg production for the poultry industry [3], whereas *Salmonella* cause various diseases, including fowl typhoid and pullorum disease, which also result in high mortality for chicks [4]. Although the abuse of antibiotics in the poultry industry has raised great concern, and many countries have made a series of efforts to control antibiotic use in animals, even forbidding prophylactic use, however, in some developing countries, prophylactic doses of antibiotics are still widely used in chicks after they hatch to prevent or control bacterial infections.

The complex gut microbiota found in animals profoundly affects the physiological functions of the host, and maintaining intestinal homeostasis is critical for health and nutrient absorption [5–8]. Widespread antibiotic treatment of agricultural animals leads to dysregulation of their intestinal microbiota [9, 10]. Furthermore, accumulating evidence suggests that antibiotic-driven gut dysbiosis may potentially increase the host’s susceptibility to some diseases and impair antibody responses [11, 12]. An intact microbiota has been reported to efficiently limit avian influenza virus (AIV) replication in ducks [13]. Evidence has shown that the establishment of the neonatal microbiota promotes the development of the immune system in the gut, and other parts of the body, for defense against pathogens [14–17]. In addition, extensive use of antibiotics in livestock has been implicated in the proliferation of antibiotic resistance genes (ARGs) in animals [8, 18, 19], which pose serious public health risks. For example, feces from chickens treated with the antibiotic chlortetracycline present a higher abundance of tetracycline resistance genes compared to those without chlortetracycline treatment [20]. Antibiotic treatment has also been reported to promote the abundance and diversity of ARGs in the microbiome of medicated swine [10].

Despite the several benefits to productivity in poultry farming, the potential side effects of prophylactic antibiotics administration on chickens have been neglected [21]. Although many developed countries have banned the use of critically important antibiotics in animal production, antibiotics are still broadly administered in poultry farms, particularly in developing countries [22]. Though substantial research has been focused on understanding the effects of therapeutic antibiotics on gut microbiota, few studies have investigated the longitudinal effect of prophylactic antibiotic use on the intestinal microbiome of chickens, as well as the collateral influence of this treatment on host resistance against viral infections. Given that the gut microbiota in early-life imprints the host immune phenotype for a long period of time and affects the ability to resist disease in later phases of life [23, 24], investigation of the impact of early-life prophylactic use of antibiotics on intestinal microbiota and disease resistance demands extensive attention. In this study, we systematically studied the effects of two commonly used prophylactic antibiotics, enrofloxacin and florfenicol, given to chickens at different stages of growth and different durations of time, on growth performance, intestinal microbial flora, accumulation of ARGs, and resistance to H9N2 avian influenza virus (AIV) infection in these chickens.

## Results

### Later prophylactic antibiotic treatments have a better growth-promoting effect

**Fig. 1:**
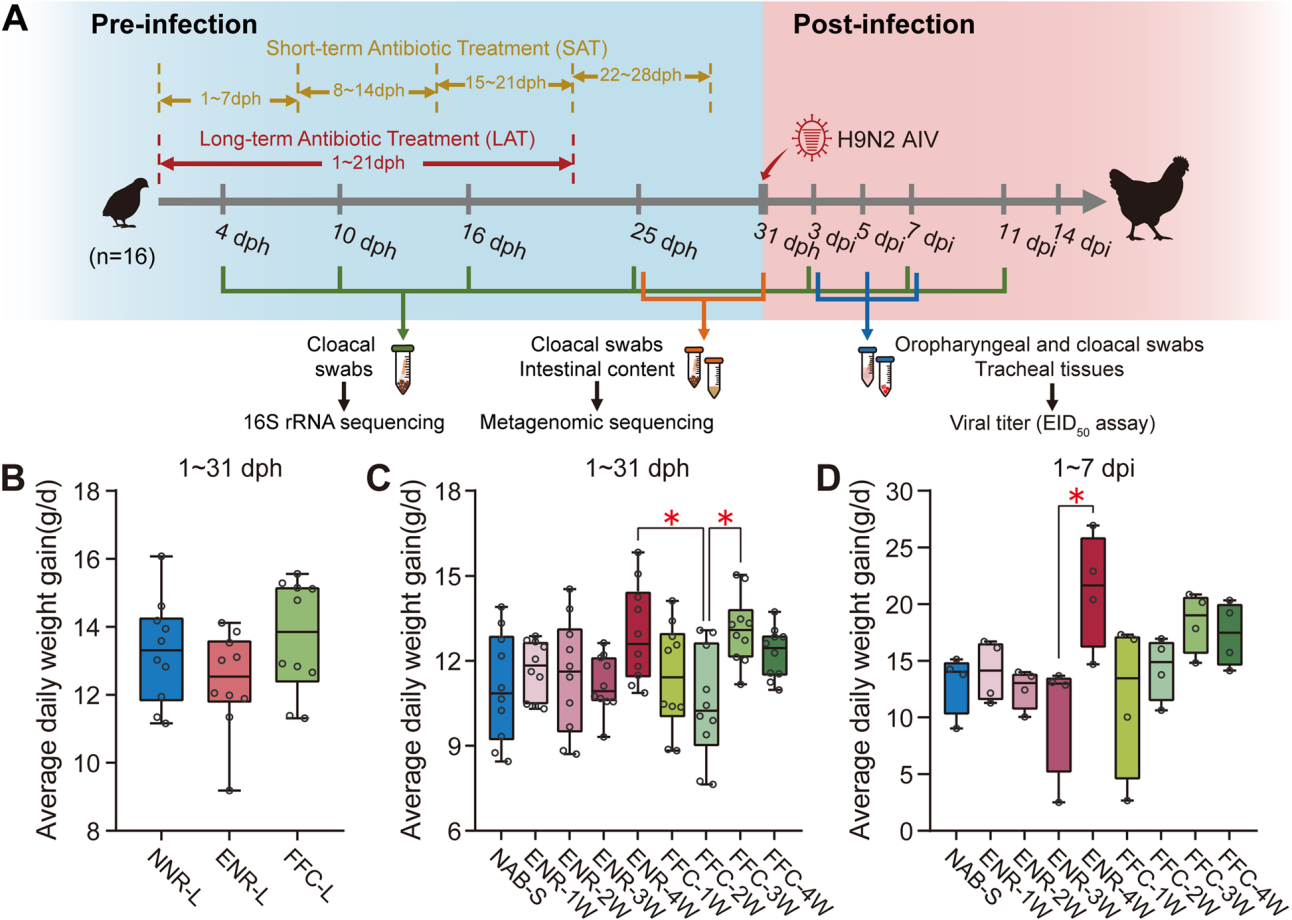
Growth performance of broiler chickens with long-term and short-term antibiotic treatment trials. **A** Schematic representation of the study design. Long-term antibiotic treatment (LAT) and short-term antibiotic treatment (SAT) trials were conducted in this study. Chickens were exposed to prophylactic antibiotic treatment from 1 to 21 dph in the LAT trial while 1 ~ 7 (1 W), 8 ~ 14 (2 W), 15 ~ 21 (3 W), and 22 ~ 28 (4 W) dph in the SAT trial. At 31 dph, all groups of chickens were infected with H9N2 AIV. Cloacal swabs were collected at seven time points, 4, 10, 16, and 25 dph (before H9N2 AIV infection) and 3, 7, and 11 dpi (after H9N2 AIV infection), to perform 16S rRNA sequencing. Cloacal swabs and intestinal content were collected at 25 and 31 dph to perform whole-metagenome shotgun sequencing, respectively. After H9N2 AIV infection, oropharyngeal swabs, cloacal swabs, and tracheal tissues were collected at 3, 5, and 7 dpi to determine viral titer. Boxplot showing average daily weight gain of chicks in the LAT (**B**), SAT (**C**) treatments, and after H9N2 AIV infection (**D**). Data was generated from ten chickens randomly selected from each group (*P* < 0.05, ANOVA, Tukey HSD test)

We recorded the average daily weight gain (ADG) to investigate whether prophylactic antibiotic treatment improved the growth performance of chickens. In the long-term antibiotic treatment (LAT) trial, chickens in the FFC-L group exhibited 3.2 and 10.4% higher ADG than those in the control and ENR-L groups, respectively, while ENR-L group exhibited 6.5% lower ADG than that the control group during the 1–31 days post-hatching (dph) period, but none of these differences was statistically significant (Fig. 1B). In the short-term antibiotic treatment (SAT) trial, except the FFC-2W group, the antibiotic-exposed chickens in all SAT groups had a higher ADG compared to the nonantibiotic-treated chickens, with the increases seen in the ENR-4W and FFC-3W groups being statistically significantly higher than in the FFC-2W group (*P* < 0.05, ANOVA, Tukey HSD test) (Fig. 1C). After H9N2 avian influenza virus (AIV) infection, chickens treated with prophylactic antibiotics in the third- or fourth-week post-hatching grew faster during first 7 days postinfection (dpi). When compared to the ENR-3W group, the growth rate of chickens in the ENR-4W group was significantly higher (*P* < 0.05, ANOVA, Tukey HSD test) (Fig. 1D). During 1 ~ 14 dpi, the later treatment groups (such as the ENR-4W, FFC-3W, and FFC-4W groups) still showed a slightly better growth performance (Supplementary Fig. 1A). To evaluate the effect of prophylactic antibiotics on immune enhancement, we also measured the immune organ index, which indicates the development of immune organs of chickens at 31 dph [25]. The long-term sub-therapeutic dose of enrofloxacin elicited a substantial increase in the bursal index compared to the control and florfenicol groups (*P* < 0.05, ANOVA, Tukey HSD test), whereas florfenicol did not show an evident effect on stimulating the growth of either the spleen or the bursa (Supplementary Fig. 1B). However, results of the SAT trial showed that 7-day antibiotic treatment (whether enrofloxacin or florfenicol) did not significantly affect the development of immune organs, indicating that the influence of short-term antibiotic exposure on immune system may be weaker (Supplementary Fig. 1C).

### Long-term prophylactic antibiotic treatments disturb the stability and functions of the chicken gut microbiota

After filtering low quality data, an average of 43,665 high-quality reads were obtained per sample in the LAT trial (Supplementary Table 2). Rarefaction curves showed that OTU richness in all samples approached saturation, suggesting that the sequencing coverage was sufficient (Supplementary Fig. 2A). To evaluate the similarity of the bacterial communities among the groups, principal coordinate analysis (PCoA) was performed using the Bray–Curtis distance matrix. The results of PCoA suggested that the divergence of the samples from three group became more distinct with an increase in medication time (Supplementary Fig. 2B & C, Supplementary Table 3). Moreover, after H9N2 virus challenge, the antibiotic groups were significantly separated from the control group (*P* = 0.001, PERMANOVA test) (Supplementary Fig. 2D, Supplementary Table 3).

We characterized the bacterial compositions to further reveal differences in the microbial communities among the LAT trial groups. Overall, at the phylum level, a total of 7 phyla including Firmicutes, Proteobacteria, Bacteroidetes, Actinobacteria, Cyanobacteria, Fusobacteria, and Verrucomicrobia were observed, with the phyla Firmicutes, Proteobacteria, and Bacteroidetes being the predominant phyla and accounting for more than 95% of the total microbial community throughout the period of this experiment (Supplementary Fig. 3A). At the genus level, a total of 154 genera were identified, with the four most abundant genera being *Escherichia-Shigella*, *Lactobacillus*, unclassified Peptostreptococcaceae, and *Bacteroides* (Supplementary Fig. 3B). Microbial composition analyses also showed differential relative abundance of taxa in the different treatment groups. For example, we found that the Firmicutes/Bacteroidetes ratio in the antibiotic groups varied more dramatically compared to the control group in the microbial maturation stage, especially in the ENR-L group, which had a significantly higher Firmicutes/Bacteroidetes ratio than the FFC-L group at 25 dph (*P* < 0.05, ANOVA, Tukey HSD), indicative of drastic changes in the gut microbiota after antibiotic exposure (Supplementary Fig. 4A). Additionally, we observed a total of 34 divergent genera with significant differences between the three groups (*P* < 0.05, ANOVA, Tukey HSD) (Supplementary Fig. 4B).

After H9N2 infection, more than half of the phyla (4/7), Firmicutes, Proteobacteria, Bacteroidetes, and Cyanobacteria were observed to have pronounced differences across the three groups (*P* < 0.05, ANOVA, Tukey HSD) (Supplementary Fig. 5A). At the genus level, unclassified Peptostreptococcaceae in the control group at 3 dpi, and *Lactobacillus* in the ENR-L group at 11 dpi, increased significantly (*P* < 0.001, ANOVA, Tukey HSD) (Supplementary Fig. 3B, Supplementary Fig. 5B). In addition, 41 other genera displayed significant differences during the AIV infection experiment, further demonstrating the impact of long-term prophylactic antibiotics on intestinal microbes (*P* < 0.05, ANOVA, Tukey HSD) (Supplementary Fig. 5B).

**Fig. 2:**
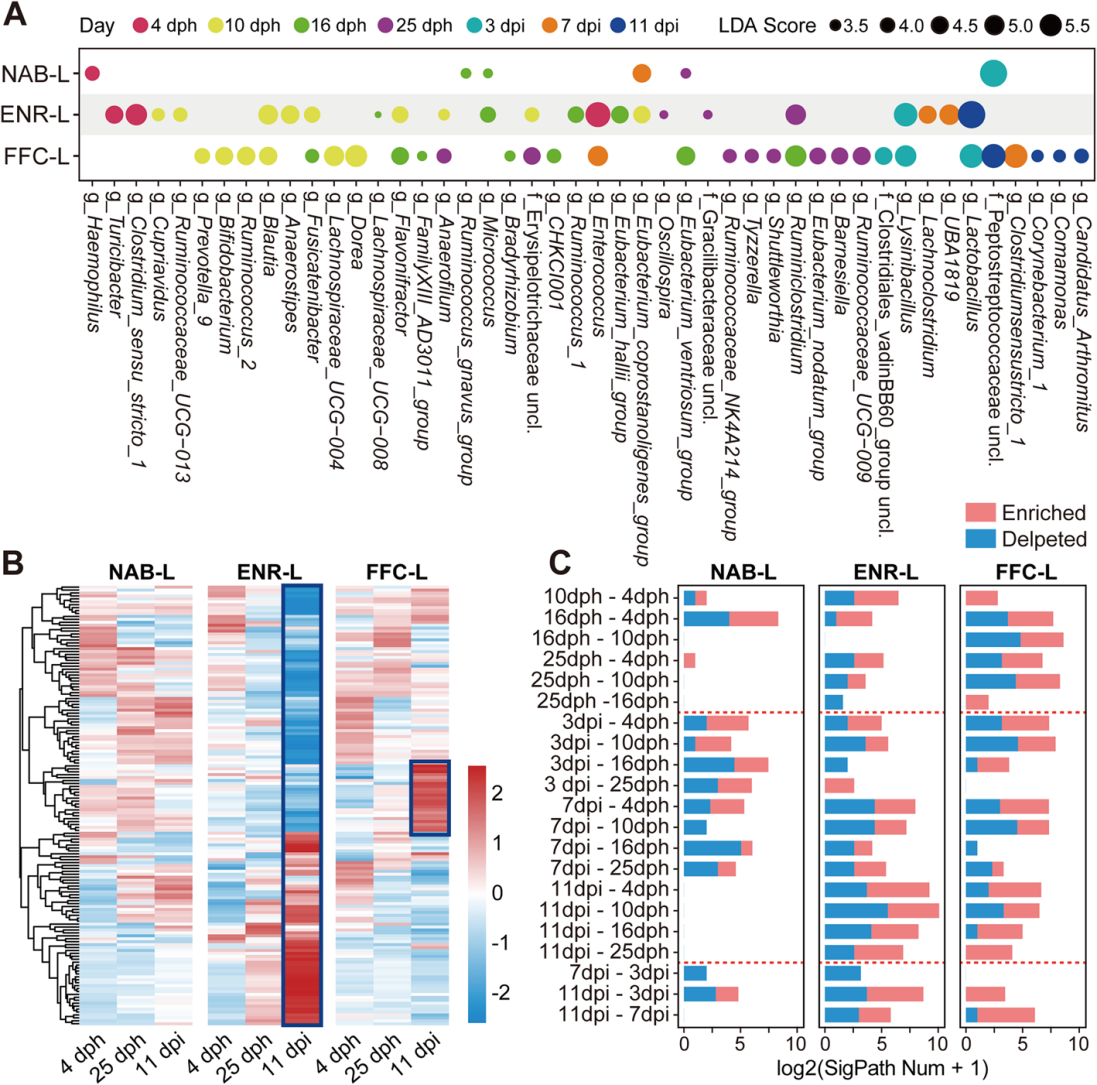
Microbial genera and the functions of the gut microbiota in chickens in the long-term antibiotic treatment trial. **A** Dot plots showing the differentially abundant genera, determined by LEfSe, between the seven sampling time points for each group (*P* < 0.05, LDA score > 2). **B** Heat maps of microbial metabolic pathways at 4 dph, 25 dph, and 11 dpi. **C** Numbers of significantly different microbial metabolic pathways, determined by DESeq2 package, in each group between the seven time points (adjusted *P* < 0.05, FoldChange > 2). SigPath Num means the number of pathways with significant difference

Linear discriminant analysis effect size (LEfSe) was performed to identify differentially abundant taxa among the seven sampling time points for each group in the LAT trial. Strikingly, we found 6, 23, and 30 different microbial genera in the NAB-L, ENR-L, and FFC-L groups, respectively (*P* < 0.05, LDA score > 2) (Fig. 2A), indicating that the gut microbiota of chickens treated long-term with antibiotics were more disturbed before and after H9N2 AIV infection. PICRUSt2 was used to predict the metagenomic functions of the microbiota, and the top 150 most abundant microbial metabolic pathways at 4 and 25 dph and 11 dpi were selected to plot heat maps. These results revealed that the functional capacity of microbiota in the antibiotic groups at 11 dpi was substantially different from that at 4 dph, particularly in the ENR-L group. In contrast, no obvious change was observed in the control group (Fig. 2B). In order to further investigate the variations in microbial metabolic pathways during the trial period, we compared the functional pathways between the seven time points in each group and counted the number of pathways with significant differences. We found that substantial bacterial functional pathways changed dramatically in the two antibiotic groups, whereas the control group’s metabolic pathway altered only slightly before AIV infection and reverted to pre-infection levels at 11 dpi. Our data provides evidence that gut microbiota without any pronounced disruption by antibiotics has a more stable metabolic function (Fig. 2C).

### Early-life prophylactic antibiotic treatment results in a more vulnerable gut microbiota in chicks

**Fig. 3:**
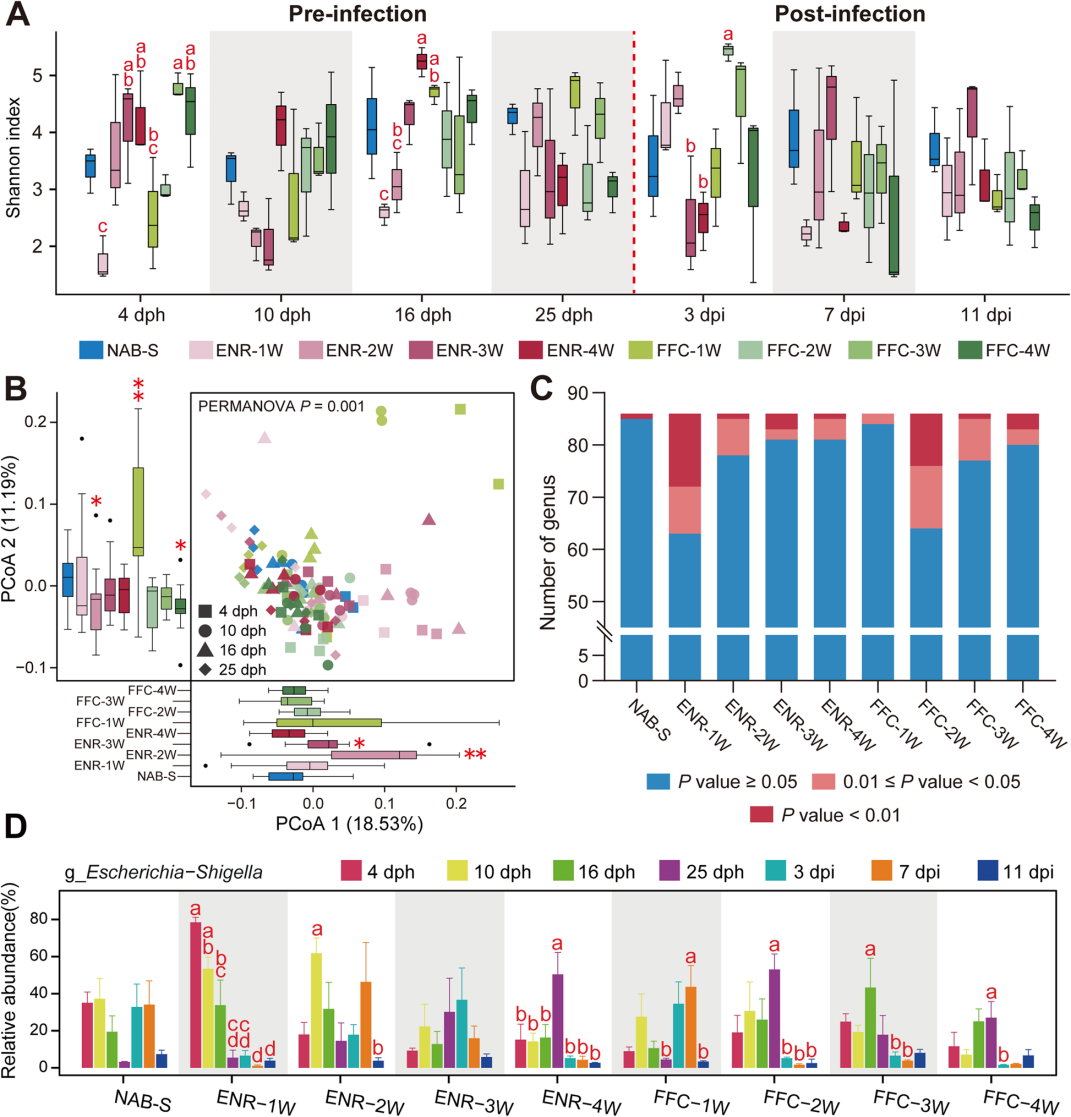
Changes in the diversity and composition of gut microbiota in chickens with time in the short-term antibiotic treatment trial. **A** Boxplot showing the Shannon diversity index of gut microbiota in chickens with time. Different red lowercase letters denote statistical significance (*P* < 0.05, ANOVA, Tukey HSD test). **B** Principal coordinate analysis (PCoA) of the bacterial communities based on Bray–Curtis distances for the pre-infection samples from the SAT trial. Below and left boxplots show the overall distribution of PCoA 1 and PCoA 2 scores within each group, and the red asterisk (*) indicates significant difference compared to the NAB-S group (*P < 0.05, **P < 0.01, Wilcoxon rank-sum test). **C** Number of bacterial genera with significant changes in each group after viral infection (ANOVA, Tukey HSD test). **D** Variation of the relative abundance of *Escherichia-Shigella* in each group. Data are represented as mean ± SE. Different lowercase letters denote statistical significance (*P* < 0.05, ANOVA, Tukey HSD test)

To further examine whether short-term administration of antibiotics at different ages results in similar disruptions in the gut microbiota in chickens, we established nine groups for a short-term antibiotic treatment (SAT) trial. A total of 189 cloacal swab samples from the SAT trial were used for 16S rRNA sequencing, yielding an average of 74,252 high-quality reads per sample after filtering (Supplementary Table 4). Rarefaction analysis revealed that the detected bacterial species reached the saturation stage. Boxplots showed that the early-stage antibiotic-fed groups had lower OTUs richness, particularly the ENR-1W and FFC-1W groups (Supplementary Fig. 6A). Before H9N2 exposure, the Shannon diversity index of several antibiotic groups, including the ENR-1W, ENR-2W, ENR-4W, FFC-1W, and FFC-4W groups, decreased in the corresponding antibiotic treatment periods. After H9N2 infection, generally, various treatment groups (except the ENR-3W group) tend to have a lower Shannon diversity than the control group at 7 and 11 dpi, although there was no significant difference (Fig. 3A); meanwhile, the Chao 1 diversity of the antibiotic groups fluctuated more drastically than the control group throughout the period of the experiment, especially the FFC-1W group at 4 dph (*P* < 0.05, ANOVA, Tukey HSD test) (Supplementary Fig. 6B). These results signify that short-term antibiotic treatment impaired the diversity and richness of the gut microbiota. Beta-diversity analysis exhibited that the majority of the pre-infection samples formed a cluster, but that many samples from the early-stage antibiotic-treated groups (the ENR-1W, ENR-2W, and FFC-1W groups) were separated from this cluster. Our data emphasize that early-stage prophylactic antibiotics had a more evident impact on the intestinal microbiota (Fig. 3B). Consistent with alpha diversity, the postinfection samples in the NAB-S group were distributed closer along the first and second principal coordinates, than those in the antibiotic groups (*P* = 0.001, PERMANOVA test; Wilcoxon rank-sum test) (Supplementary Fig. 6C).

A total of 5 phyla and 86 genera were detected based on the 16S rRNA data of the SAT trial. Similar to the LAT trial, Firmicutes, Proteobacteria, and Bacteroidetes were the three most dominant phyla, followed by Actinobacteria and Tenericutes, but the latter was only observed in the SAT trial (Supplementary Fig. 7). However, bacterial taxa identified at the genus level were quite different among the different groups, and the microbial communities were noted to be altered after viral infection (Supplementary Fig. 8). Hence, to further explore the alteration after H9N2 challenge in chickens’ gut microbiome, the numbers of the 86 bacterial genera with significant change were recorded. These results revealed that the gut microbiota in chickens with later-stage antibiotic treatment appeared to remain more stable when exposed to H9N2 AIV, implying a profound impact of early-life antibiotic treatment (Fig. 3C). Unexpectedly, expansions of *Escherichia-Shigella* were observed in the various antibiotic groups (ENR-1W and all groups of FFC short-term treatment) through our LEfSe analysis (Supplementary Fig. 9). The profiles for *Escherichia-Shigella* further displayed their pronounced increase under antibiotic treatment, demonstrating that antibiotics may provide a window for the expansion of opportunistic pathogens (Fig. 3D).

### Prophylactic antibiotics may increase susceptibility of chickens to H9N2 avian influenza virus

**Fig. 4:**
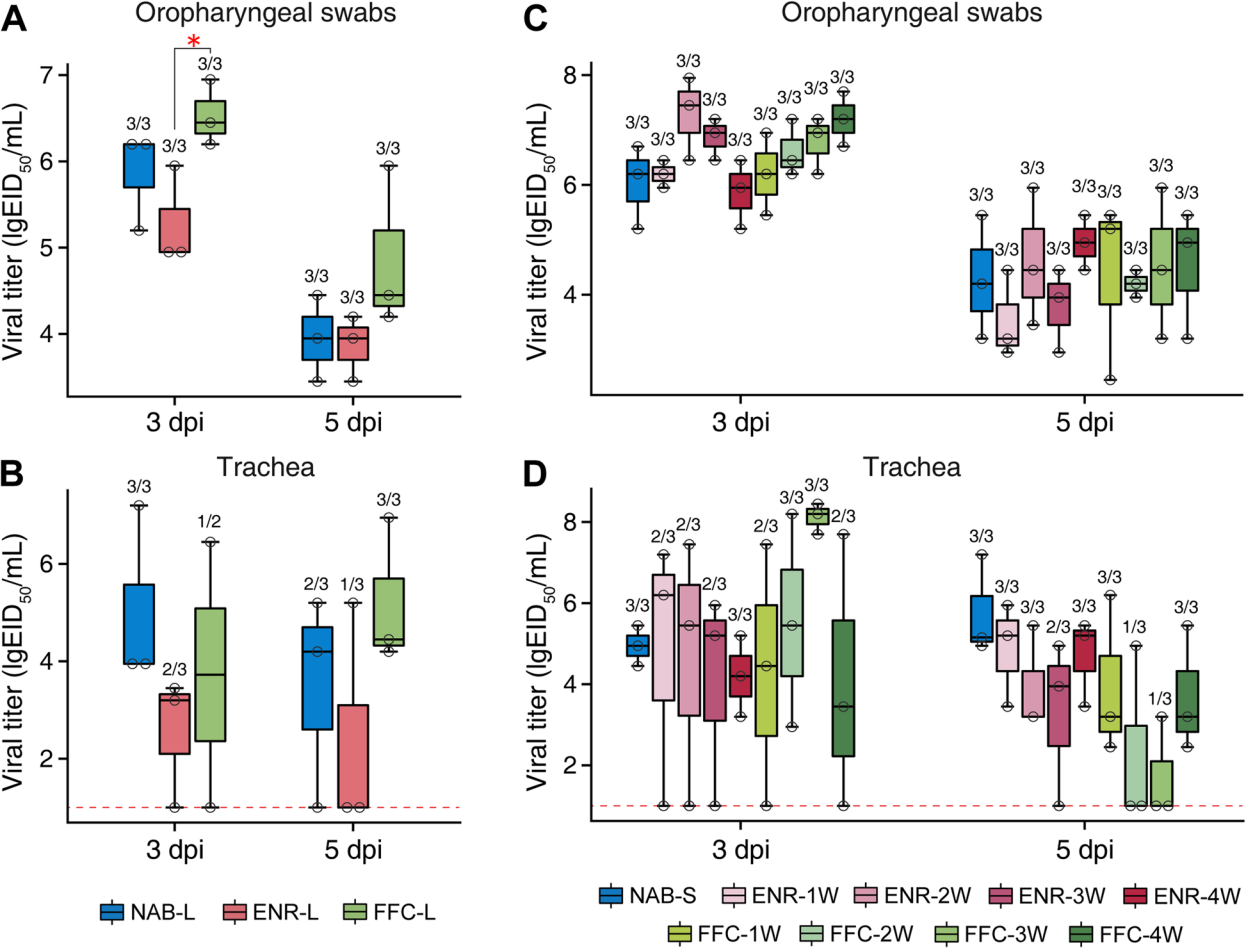
Viral titers from oropharyngeal swabs and trachea tissue from inoculated chickens in both the long-term and short-term antibiotic treatment trials. Viral titers in oropharyngeal swabs and tracheal tissues of H9N2-infected chickens in the long-term antibiotic treatment trial (**A** and **B**) and short-term antibiotic treatment trial (**C** and **D**). Ratio of positive samples to the number of all tested samples is shown above each group. Dashed black lines indicate the lower limits of detection (*P < 0.05, Kruskal–Wallis and Dunn’s tests)

During the infection experiment, inoculated chickens exhibited clinical symptoms such as depression and sternutation, but none of the chickens died. In the LAT trial, viral titers in the oropharyngeal swabs were highest in FFC-L group at both 3 and 5 dpi, with mean titers of 6.65 and 5.49 lgEID_50_/mL, respectively. In particular, the viral load in the oropharyngeal swabs from the FFC-L group was significantly higher at 3 dpi compared to the ENR-L group (*P* < 0.05, Kruskal–Wallis and Dunn’s tests, Fig. 4A). Viral load in the oropharyngeal swabs and tracheal tissue was higher in chickens with 21-day florfenicol treatment (FFC-L group) at 5 dpi, but the difference was not statistically significant (Fig. 4B). Additionally, the SAT trial results revealed that viral titers in a number of antibiotic groups were higher than in the control group (NAB-S) at 3 dpi. For example, in oropharyngeal swabs, viral load in the chickens from the antibiotic groups, excluding ENR-1W and ENR-4W group, was increased compared to the control group (Fig. 4C). Similar to oropharyngeal results, we detected higher viral titers in trachea tissue in the antibiotic groups, except for the ENR-4W group, although these increases were not statistically significant (Fig. 4D). We further measured cytokine levels in the pre- and postinfection serum of chickens (Supplementary Fig. 10). Our results showed that long-term prophylactic enrofloxacin exposure decreased serum cytokines levels, particularly IL-1β and IL-18 at 31 dph (*P* < 0.05, ANOVA, Tukey HSD test) (Supplementary Fig. 10A). Interestingly, short-term antibiotic treatment did not result in similar significant reductions before infection. Moreover, early-life prophylactic antibiotic treatment groups (ENR-1W, ENR-2W) had higher IL-1β at 14 dpi (Supplementary Fig. 10B).

### Prophylactic antibiotics expanded the resistome in the chicken gut microbiota

**Fig. 5:**
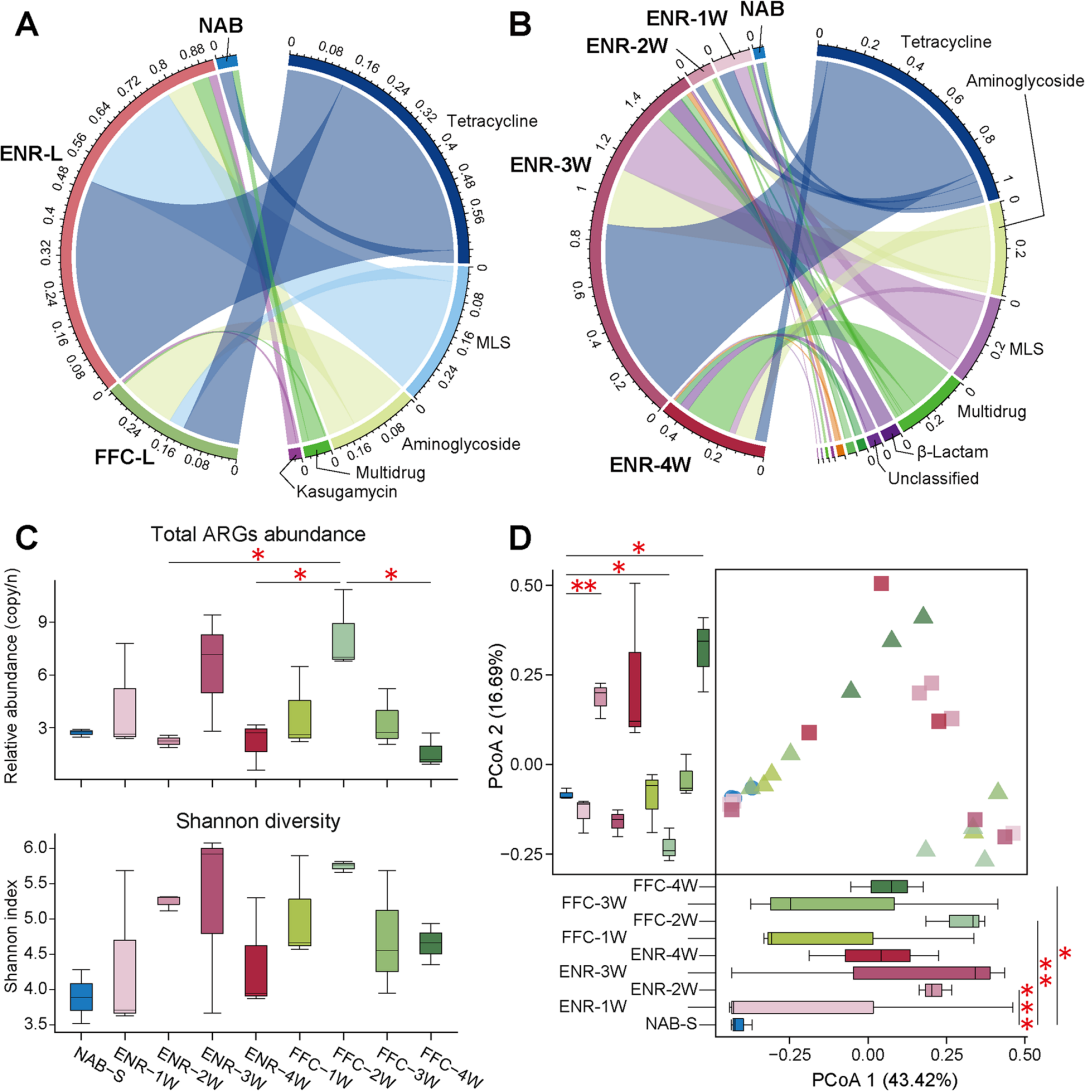
Effect of prophylactic antibiotic treatment on ARG composition and diversity of the chicken gut microbiome. Chord diagrams showing the relative abundance of ARGs in the intestinal contents collected from **A** the LAT and **B** SAT trials. The outmost circle lists the names of intestinal content samples and detected ARG types. The connecting lines inside the circle links ARG types to samples, and the width of the lines is proportional to the relative abundance (%) of each ARG type in the corresponding sample. **C** Box plots showing total ARGs abundance and Shannon diversity index of the resistome in the cloacal swabs collected from the SAT trial at 25 dph (*P < 0.05, ANOVA, Tukey HSD test). **D** PCoA plot of the ARG communities in the cloacal swabs collected from the SAT trial at 25 dph based on the Bray–Curtis distances. Below and left boxplots show the overall distribution of PCoA 1 and PCoA 2 scores within each group, and the red asterisk (*) indicates significant difference compared to the NAB-S group (*P < 0.05, **P < 0.01, Wilcoxon rank-sum test)

The intestinal resistome contents were characterized and quantified from the metagenomic data using ARGs-OAP. Based on the SARG database, we detected an average of 0.52 ARG copies per cell in the intestinal content, including a total of 13 ARG types and 72 ARG subtypes (Supplementary Table 6). The most dominant ARG types in the gastrointestinal samples were tetracycline, aminoglycoside, macrolide-lincosamide-streptogramin (MLS), and multidrug resistance genes. Long-term antibiotic treatment induced an enrichment of antimicrobial resistance genes in the gut microbiome (Fig. 5A). Although the time of antibiotic exposure was shortened, the levels of ARGs in the intestinal microflora of the SAT groups were still increased (Fig. 5B). To further investigate the resistome in chicken excretions, we performed a metagenomic analysis of the cloacal swabs collected from the SAT trials. In total, 158 ARG subtypes from 16 ARG types were detected in the cloacal swabs, with an average of 3.86 ARG copies per cell, which is substantially different from the gastrointestinal samples, including abundance and diversity of the resistome (Supplementary Table 6). Our findings reveal that, compared to the control group, the total abundance of ARGs was elevated after antibiotic exposure, especially in the FFC-2W group, where ARG abundance was significantly higher than that in several antibiotic groups (such as the ENR-2W, ENR-4W, and FFC-4W groups) (*P* < 0.05, ANOVA, Tukey HSD test, Fig. 5C). The composition profile showed that the predominant ARG types in the cloacal swabs were multidrug, aminoglycoside, tetracycline, and MLS resistance genes. The relative abundance of multidrug resistance genes seemed to be strongly associated with antibiotic treatment (Supplementary Fig. 11). Additionally, PCoA exhibited that the structure of the resistance-genes was altered markedly by antibiotics (Fig. 5D).

## Discussion

The commercial poultry industry relies on raising large quantities of birds at high stocking densities and see the use of antibiotics as beneficial for increased feed conversion, growth promotion, and disease prevention [26]. Prophylactic doses of antibiotics are widely used on newborn chicks to control bacterial infection in many developing countries. In this study, we systematically investigated the effect of prophylactic antibiotics on growth performance, gut microbiota, and the levels of antibiotic resistance genes in the gut microbial community. In addition, we evaluated the resistance of chickens to H9N2 AIV following a long-term or a short-term early-life prophylactic antibiotic treatment.

In contrast to the traditional expectation, we concluded from our results that long-term prophylactic enrofloxacin treatment does not accelerate growth rate, and, instead, it has the opposite effect (Fig. 1B). However, chickens treated with a prophylactic antibiotic at a later-stage (15 ~ 28 dph) exhibited more rapid growth both before and after influenza virus infection compared to earlier-stage treatments (Fig. 1B, C, & D). The Firmicutes/Bacteroidetes ratio was higher in the antibiotic treatment groups (Supplementary Fig. 4A). Firmicutes bacteria play a key role in nutrition and metabolism of the host animal through short-chain fatty acid synthesis. An increased Firmicutes/Bacteroidetes ratio is associated with obesity [27]. This may explain the better growth performance of most antibiotic groups. Furthermore, chickens that had antibiotic exposure at earlier stages (1 ~ 14 dph, 1 W and 2 W groups) showed disruptions in their gut microbial development (Fig. 3A, Supplementary Fig. 6B). The robust growth performance of animals is inextricably linked to a stable and healthy gut microbiome [28]. The indigenous microbial colonization in the gastrointestinal tract of chickens occurs primarily within 2 weeks after hatching and then gradually becomes stabilized [29, 30]. Antibiotic exposure in early stages disturbs colonization of gut by microbiota in chickens and may have a negative impact on their growth rates.

Gut-resident microbes not only affect the growth and development of their hosts [31] but also modulate collectively various host physiological activities [32]. However, gut microbiota is sensitive to a variety of disturbances [33, 34]. Especially in the developing infant, the gut microbiota is highly dynamic and prone to disruption by external factors, including antibiotic exposure [35]. Our work revealed that even though chickens were only exposed to sub-therapeutic doses of antibiotics, they showed reduction in microbial diversity. We noted changes in relative abundance of the microbial communities not only in the long-term but also in the short-term antibiotic treatment groups (Figs. 2 and 3). After H9N2 challenge, the functional capacities of the microbiota in the long-term antibiotic-treated chickens were substantially different at 11 dpi, whereas the microbial functions of non-treated chickens had nominal changes (Fig. 2B). Importantly, metagenomic functions of the microbiota in the NAB-L group quickly adjusted back to their pre-infection levels at 11 dpi, demonstrating the robust adaptation and resilience of the gut microbiota in non-treated chickens (Fig. 2C). Consistent with the results of the LAT trial, short-term antibiotic exposure also led to gut microbiota volatility after viral infection. Notably, chickens treated with earlier-stage prophylactic antibiotics were more vulnerable (Fig. 3C). Additionally, antibiotic usage provided an opportunity for an increase in the abundance of opportunistic pathogens, such as *Escherichia-Shigella* (Fig. 3D), which is contrary to our understanding of the effects of antibiotics. Thus, the harm of early-life and long-term prophylactic antibiotic exposure in flock cannot be neglected.

Natural microbiota can improve host fitness and survival to diverse disease challenges [36]. Our previous study demonstrated that early-life establishment of the gut microbiota plays a critical role in host defense against viruses [16]. Conversely, less stable intestinal microbiome might result in a decreased antiviral immune response. Our observations from the H9N2 infection experiment are consistent with this report. Antibiotic-treated chickens tend to have higher viral titers, which is consistent with the highly pathogenic H5N9 AIV infection experiments in antibiotic-exposed ducks [13], collectively highlighting the importance of an intact gut microbiota in antiviral defense. Cytokines play an essential role in the immune responses against influenza virus infection [37]. Recent studies found that following the disruption of the host gut microbiota, antibiotic treatment blocked interferon signaling and impaired mRNA expression of some cytokines, facilitating early replication by the influenza virus [4, 37, 38]. Our observations of higher viral titers and lower levels of cytokines in chickens with antibiotic treatment, compared to control chickens, are consistent with these findings. Notably, we observed that later-stage antibiotic-treated chickens had similar disease resistance and cytokines levels as non-treated chickens. Our data emphasize the impact of early-life prophylactic antibiotic exposure on chickens.

In addition, antibiotic resistance poses a substantial threat to public health, and the widespread use of antibiotics in agricultural production has raised public concern [26, 39]. In this study, our results indicate that antibiotic treatment expanded the resistome in gut microbiota, even with a 7-day sub-therapeutic antibiotic exposure. Moreover, we observed that antibiotic treatment altered the structure of the resistome in chicken feces. Caution should be exercised while administering prophylactic antibiotics in poultry farms.

## Conclusions

Prophylactic antibiotics have growth-promoting effects and are widely used to control mortality associated with bacterial infections in neonatal poultry. Our findings provide evidence that long-term prophylactic enrofloxacin treatment slows the growth rate, whereas short-term use of antibiotics may increase growth rate in chickens. However, early-life administration of prophylactic antibiotics disturbs the establishment and reduces the stability of the gut microbiota in chickens, with concomitant increases in the abundance of opportunistic pathogens, such as *Escherichia-Shigella*, and has higher viral titers after H9N2 AIV infection. Furthermore, our data reveal that chickens exposed to either long-term or short-term prophylactic antibiotics expand their gut microbiota resistome, thus posing a serious threat to public health. Our findings highlight the threat of prophylactic antibiotics treatment and provide a theoretical basis for the cautious administration of antibiotics in food-producing animal management.

## Methods

### Animals and experimental design

In the long-term antibiotic treatment (LAT) trial, 48 post-hatching specific-pathogen-free (SPF) chicks were randomly placed into three groups. All chicks were provided free access to base diet and water. In previous studies, antibiotic treatments were administrated by adding antibiotics to diet or drinking water [1, 40, 41]. In this study, from days 1 to 21 post-hatching (dph), two of the groups were treated with either enrofloxacin (ENR-L) or florfenicol (FFC-L) through their drinking water, using a prophylactic antibiotic dose of 75 mg/L. The control group (NAB-L) was not treated with antibiotics. Enrofloxacin and florfenicol were chosen because they are two broad-spectrum antibiotics that are widely used for prophylactic treatment as well as therapeutic treatment in the poultry industry in China.

To investigate the impact of administration of antibiotics in different life stages, we performed a short-term antibiotic treatment (SAT) trial. In this trial, 144 post-hatching chicks were randomly placed into nine groups. The antibiotic treatment groups were exposed to either enrofloxacin or florfenicol for four different time periods, days 1–7, days 8–14, days 15–21, or days 22–28 post-hatching. Thus, the SAT trial had nine groups: enrofloxacin for 1–7, 8–14, 15–21, and 22–28 dph (ENR-1W, ENR-2W, ENR-3W, and ENR-4W, respectively); florfenicol for 1–7, 8–14, 15–21, and 22–28 dph (FFC-1W, FFC-2W, FFC-3W, and FFC-4W, respectively); and a no antibiotic control group (NAB-S) (Supplementary Table 1).

For chicks of all groups, including the LAT and SAT trials and the control group, cloacal swabs were collected at 4, 10, 16, and 25 dph. The weight of ten chickens in each group was individually monitored every 3 days from 1 to 31 dph, with feed removed for 12 h before each weighing. At 31 dph, five chickens from each group were randomly selected and were euthanized before H9N2 avian influenza virus (AVI) challenge. Spleen and bursa of each chicken were collected and weighed to calculate an immune organ index that was defined as the spleen (or bursal) weight (mg) divided by the overall body weight (g). Simultaneously, intestinal contents and blood were aseptically collected. Serum was prepared from the blood and stored at − 80 °C.

### H9N2 avian influenza virus infection experiments

To assess the impact of prophylactic antibiotics on disease resistance, chickens in all groups, including the LAT and SAT trials and the control group, were inoculated intranasally and intraocularly with H9N2 AIV. H9N2 AIV (strain name: A/chicken/Guangdong/Lz-wzp-10/2013, GenBank accession numbers OK035258 to OK035265) was propagated using 10-day-old SPF embryonated chicken eggs. The allantoic fluid collected at 72-h post-inoculation was titrated using the Reed-Muench method [42]. At 31 dph, 11 chicks from all groups were challenged with 10^6^ EID_50_ of H9N2 AVI in a volume of 200 μL via the ocular and nasal routes. To determine the level of viral replication for each group, oropharyngeal and cloacal swabs of the chickens were collected and suspended in 1-mL Dulbecco’s Modified Eagle’s Medium (DMEM) with antibiotics (penicillin and streptomycin, 10,000 U/mL) at 3, 5, and 7 days postinfection (dpi). In addition, three chickens were randomly selected from each group at 3 and 5 dpi and euthanized to collect tracheal tissues. Virus was undetectable after 7 dpi. At 14 dpi, blood was collected from the remaining chickens through their wing veins to prepare serum. All samples were stored at − 80 °C until further use. The complete study design is shown in Fig. 1A.

### Viral titration

Oropharyngeal and cloacal swabs were suspended and vibrated in 1-mL DMEM with antibiotics (penicillin and streptomycin, 10,000 U/mL). Tracheal and lung tissue samples were homogenized at 20% (w/v) in DMEM with antibiotics (penicillin and streptomycin, 10,000 U/mL). After centrifugation at 3500 rpm for 5 min at 4 °C, the supernatant was inoculated into 10-day-old SPF embryonated chicken eggs. After 72 h of inoculation, the viral titers of the samples were determined using the hemagglutination assay (HA) and calculated based on the Reed-Muench method.

### Detection of serum cytokines levels

After coagulation at room temperature for 15 min, blood samples were centrifuged at 2500 rpm for 10 min to collect their supernatants. IFN-β, IL-1β, and IL-18 levels in the serum samples were measured using ELISA assay kits (Meimian, China). Optical density (OD) values at 450 nm were measured using a RT-6100 microplate reader (Rayto, USA).

### DNA extraction and 16S rRNA sequencing

It might be possible that chicks have different water intake and lead to differences in antibiotics intake. To reduce this bias and ensure sequencing efficacy, three cloacal swabs were combined into a single composite sample according to collection date and group. 16S rRNA sequencing was performed on the composite cloacal swabs collected at 4, 10, 16, and 25 dph and 3, 7, and 11 dpi.

Total genomic DNA from the samples was extracted using the SDS method and purified through 1% agarose gels. PCR amplicons targeting the V3 and V4 hypervariable regions of the 16S rRNA gene were obtained with the primers 341F (5′-CCTAYGGGRBG CASCAG-3′) and 806R (5′-GGACTACHVGGGTWTCTAAT-3′). TruSeq DNA PCR-Free Sample Preparation Kit (Illumina, USA) were used to generate the sequencing libraries. The quantified libraries were sequenced on the Illumina NovaSeq platform to generate 250-bp paired-end reads, with an average of 100,091 ± 19,426 and 104,643 ± 11,285 paired-end reads per sample produced, respectively, in the LAT and SAT trials (further details in Supplementary Tables 2 &  3).

### 16S rRNA data processing

Raw paired-end reads were merged with FLASH (v1.2.11) software [43]. Raw tags were assigned to samples based on their unique barcodes and were quantified using Quantitative Insights into Microbial Ecology (QIIME v1.9.1) [44]. Barcode and primer sequences were removed using Cutadapt (v3.5) [45]. Based on a comparison with the reference SILVA database (v132) [46], chimeric and nonbacterial sequences were detected and removed to obtain clean tags using USEARCH (v11) [47]. The UPARSE algorithm [48] was then used to cluster all clean tags into operational taxonomic units (OTUs) with a sequence similarity of 97%, and representative sequences for the OTUs were subsequently mapped to the SILVA database to determine taxonomy. The alpha-diversity index and beta diversity were calculated from the normalized OTU table employing the QIIME pipeline.

Beta-diversity analysis was performed with principal coordinates analysis (PCoA) based on Bray–Curtis dissimilarity values. The permutational multivariate analysis of variances (PERMANOVA) was employed to calculate the significance of the differences in community compositions between the groups with 999 permutations using the vegan package implemented in R. LEfSe [49] was used to compare and identify significantly different bacterial species between each group (*P* < 0.05, LDA score > 2). PICRUSt2 [50] was used to predict the metagenomic functions of the microbiota in each group. The DESeq2 package [51] was employed to analyze differentially abundant microbial metabolic pathways using threshold criteria of adjusted *P* < 0.05 and FoldChange > 2.

### Metagenomic sequencing and assembly

Metagenomic sequencing was performed on the composite intestinal content samples collected at 31 dph and composite cloacal swabs collected at 25 dph (Supplementary Table 5). DNA was isolated with the Qiagen QIAamp DNA Stool Mini Kit (Qiagen, Germany) according to the manufacturer’s protocol. Metagenomic DNA paired-end libraries were generated with NEBNext Ultra DNA Library Prep Kit for Illumina (New England Biolabs, USA) with an insert size of 350 bp.

Metagenomic sequencing was performed on an Illumina platform, with an average of 6.66-GB raw data per sample produced. The raw reads for each sample were independently processed to filter low-quality reads using fastp (v0.23.0) [52]. Contamination reads were removed by mapping the high-quality reads to the chicken genome (NCBI Genome ID: GRCg7b) through BWA-MEM (v 0.7.17) [53] and SAMtools (v1.9) [54]. Clean reads were annotated as ARG-like reads using ARGs-OAP (v2.0) [55] with the structured ARG reference (SARG) database, which was constructed by integrating the antibiotic resistance genes database (ARDB), the Comprehensive Antibiotic Resistance Database (CARD), and NCBI-NR database. The abundance of ARGs was normalized into copy of ARGs per cell, and the cell number was computed based on the number of 16S rRNA genes. ARG types and subtypes were also counted with the algorithm implemented in the pipeline.

## Supplementary Information

**Additional file 1: Supplementary Fig. 1.** Growth performance and immune organ indexes of broiler chickens. (A) Average daily weight gains in chickens in the SAT trial during the period 1 ~ 14 days post-H9N2-infection. Immune organ indexes from spleen and bursa of five randomly selected chickens from each group were calculated at 31 dph in (B) the LAT and (C) SAT trial (* *P* < 0.05, ANOVA, Tukey HSD).

**Additional file 2: Supplementary Fig. 2.** Shifts of gut microbial similarity in samples collected from the three groups in the LAT trial. (A) Rarefaction curves generated on the observed number of OTUs. Principal coordinate analysis (PCoA) of the bacterial communities in samples collected at 4 and 10 dph (B), 16 and 25 dph (C), 3 and 7 dpi (D) based on Bray–Curtis distances. Below and left boxplots show the overall distribution of PCoA 1 and PCoA 2 scores within each group and red asterisk (*) indicates significant difference compared to the control (NAB-S) group (* *P* < 0.05, ** *P* < 0.01, Wilcoxon rank-sum test).

**Additional file 3: Supplementary Fig. 3.** Composition and relative abundance of microbial communities in the different groups in the LAT trial. Stacked bar charts show phyla (A) and the top 10 most abundant bacterial genera (B). Each color represents the relative abundance of a bacterial taxon on the stacked bar chart.

**Additional file 4: Supplementary Fig. 4.** The differences of microbial communities among three groups in the LAT trial before H9N2 AIV infection. (A) Boxplots show the Firmicutes/Bacteroidetes ratio in the three groups at 16 and 25 dph. (B) Heat maps show the genera with significant differences between the three groups at 4, 10, 16 and 25 dph (* *P* < 0.05, ** *P* < 0.01, *** *P* < 0.001, ANOVA, Tukey HSD).

**Additional file 5: Supplementary Fig. 5.** Differences in microbial communities among the three groups in the LAT trial after H9N2 AIV infection. (A) Boxplots showchanges in the four major phyla in the three groups at 3, 7 and 11 dpi. (B) Heat maps show the genera with significant differences between the three groups at 3, 7 and 11 dpi (* *P* < 0.05, ** *P* < 0.01, *** *P* < 0.001, ANOVA, Tukey HSD).

**Additional file 6: Supplementary Fig. 6.** Shifts of gut microbial diversity in chickens that received short-term antibiotic treatment. (A) Rarefaction curves generated from observed numbers of OTUs. Boxplots on the right shows the overall distribution. (B) Boxplot shows the Chao 1 diversity index of gut microbiota in chickens with time. Different red lowercase letters denote statistical significance (*P* < 0.05, ANOVA, Tukey HSD test). (C) Principal coordinate analysis (PCoA) of the bacterial communities based on the Bray–Curtis distances for postinfection samples from the SAT trial. Two outliers from the ENR-1W and FFC-2W group at 11 dpi were removed from the plot. Below and left boxplots show the overall distribution of PCoA 1 and PCoA 2 scores within each groups and the red asterisk (*) indicates significant difference compared to the NAB-S group (* *P* < 0.05, ** *P* < 0.01, Wilcoxon rank-sum test).

**Additional file 7: Supplementary Fig. 7.** Relative abundance of the bacterial phyla in the different groups in the SAT trial. Stacked bar charts show taxa at the phylum level. Each color represents the relative abundance of a bacterial phylum on the stacked bar chart.

**Additional file 8: Supplementary Fig. 8.** Relative abundance of the top 10 most abundant bacterial genera in the different groups in the SAT trial. Stacked bar charts show taxa at the genus level. Each color represents the relative abundance of a bacterial genus on the stacked bar chart.

**Additional file 9: Supplementary Fig. 9.** Strikingly different microbial genera of each group in the SAT trial. Dot plots show differentially abundant genera determined by LEfSe between the seven sampling time points in each group (*P* < 0.05, LDA score > 2).

**Additional file 10: Supplementary Fig. 10.** Levels of cytokines in the prophylactic antibiotic-treated chickens at 31dph and 14 dpi. Boxplot showing levels of IFN-β, IL-1β and IL-18 in serum collected from chickens in the LAT (A) and SAT trials (B) at 31dph and 14 dpi (* *P* < 0.05, Tukey HSD).

**Additional file 11: Supplementary Fig. 11.** Relative abundance of ARGs in the different groups in the SAT trial. Stacked bar charts show relative abundance of ARGs. Each color represents the relative abundance of an ARG on the stacked bar chart.

**Additional file 12: Supplementary Table 1.** Group setting of the study. **Supplementary Table 2.** Sequencing depth of the 16S rRNA genes for the samples in the long-term treatment trial. **Supplementary Table 3.** Results of the PERMANOVA test in the LAT trial. **Supplementary Table 4.** Sequencing depth of the 16S rRNA genes for the samples in the short-term treatment trial. **Supplementary Table 5.** Information of samples used for metagenomic sequencing. **Supplementary Table 6.** Relative abundance of ARG types.

## Data Availability

The datasets generated in the current study were deposited to the NCBISRA database under the BioProject accession no. PRJNA880549.

## Abbreviations

ADG: Average daily weight gain
AGP: Antibiotic growth promotor
AIV: Avian influenza virus
ARG: Antibiotic resistance gene
dph: Days post-hatching
dpi: Days postinfection
LAT: Long-term antibiotic treatment
LEfSe: Linear-discriminant analysis effect size
OTU: Operational taxonomic unit
PCoA: Principal coordinates analysis
PERMANOVA: Permutational multivariate analysis of variances
SAT: Short-term antibiotic treatment
