# Supplementary figures and images for: Early-life prophylactic antibiotic treatment disturbs the stability of the gut microbiota and increases susceptibility to H9N2 AIV in chicks
Source: Microbiome. 2023 Jul 26;11:163. doi: 10.1186/s40168-023-01609-8 (PMC10369819; doi:10.1186/s40168-023-01609-8)

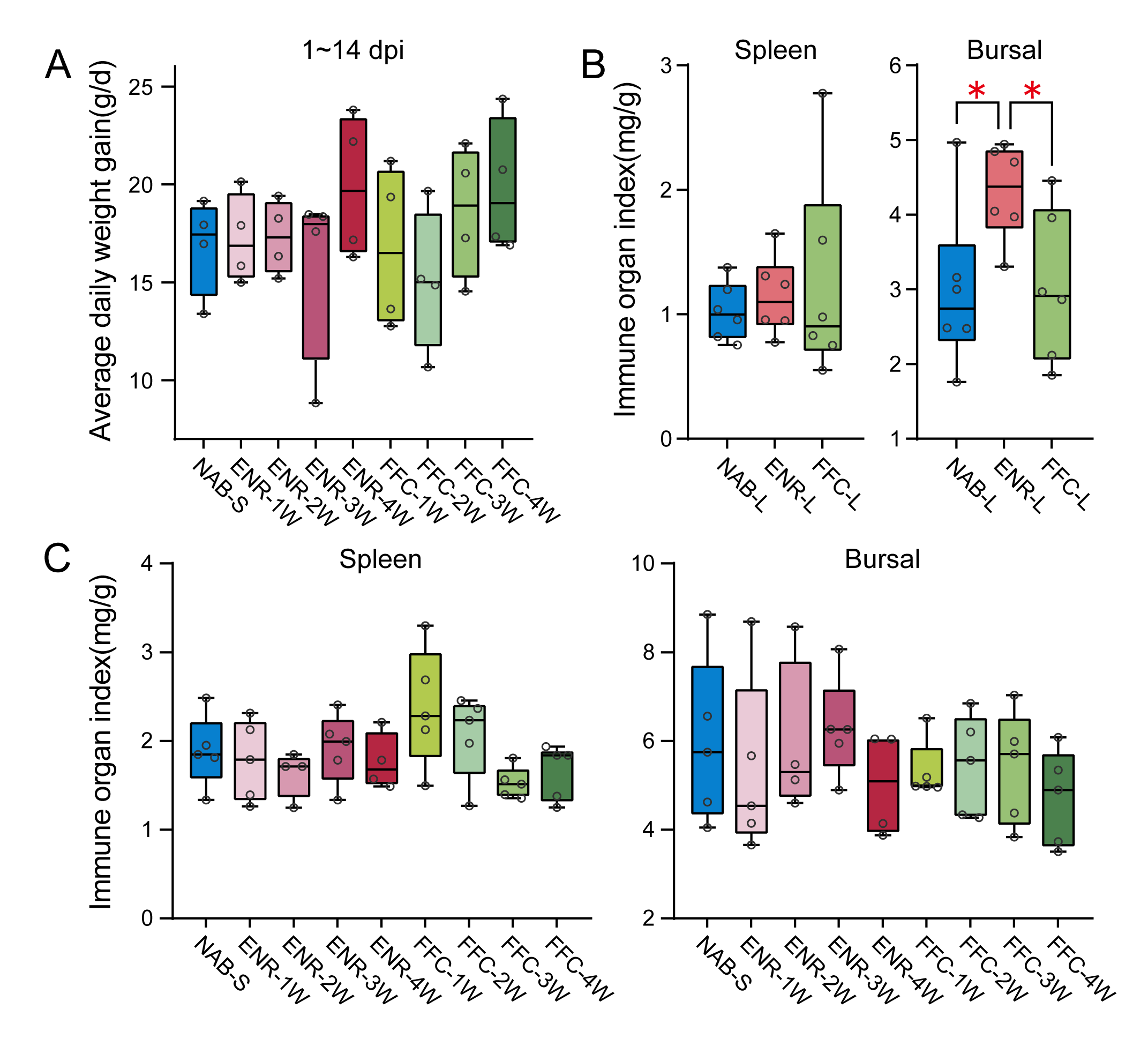

Supplement: Supplementary file 2 — Additional file 1: Supplementary Fig. 1. Growth performance and immune organ indexes of broiler chickens. (A) Average daily weight gains in chickens in the SAT trial during the period 1 ~ 14 days post-H9N2-infection. Immune organ indexes from spleen and bursa of five randomly selected chickens from each group were calculated at 31 dph in (B) the LAT and (C) SAT trial (* P < 0.05, ANOVA, Tukey HSD). [file 40168_2023_1609_MOESM1_ESM.tif]

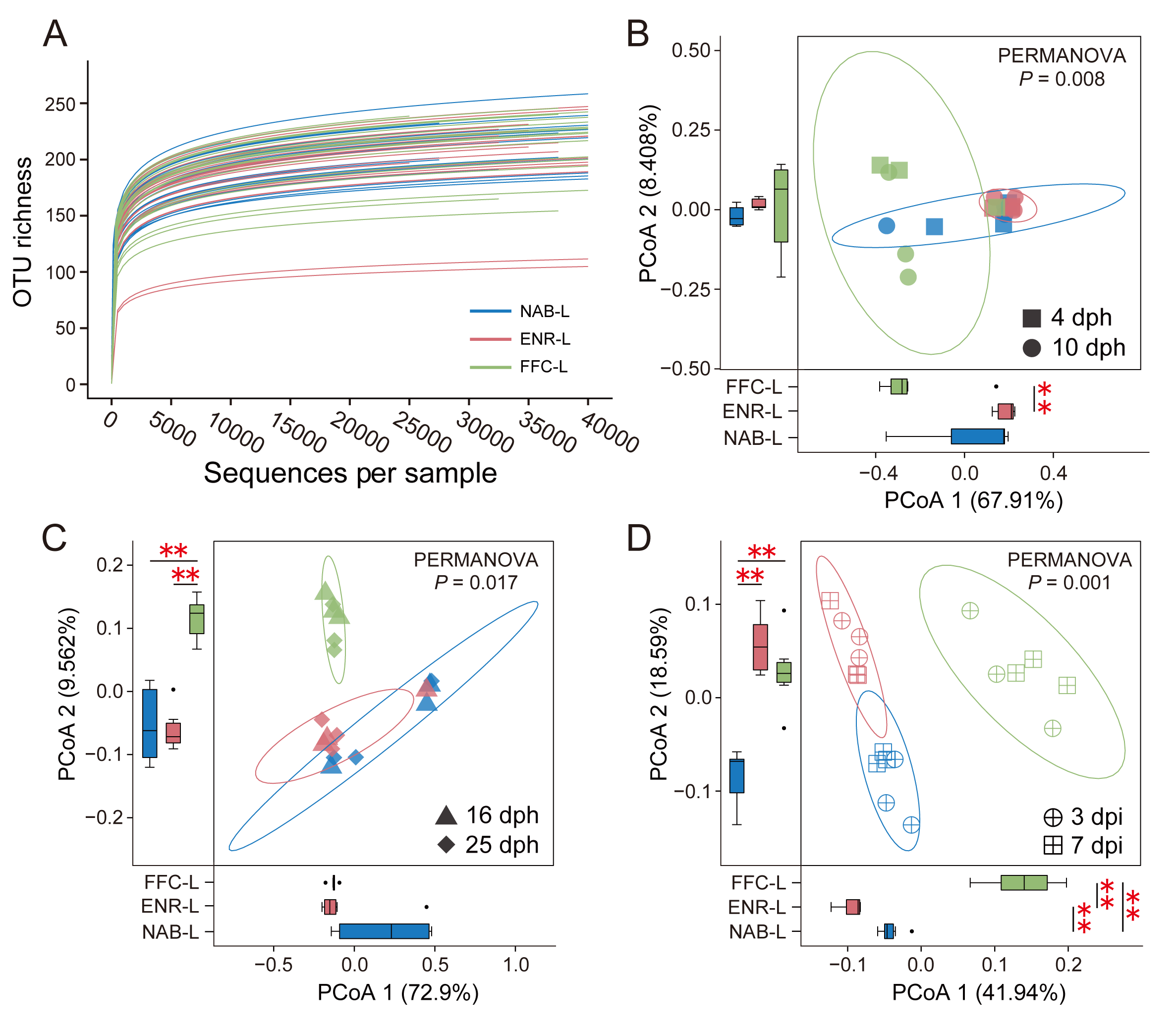

Supplement: Supplementary file 3 — Additional file 2: Supplementary Fig. 2. Shifts of gut microbial similarity in samples collected from the three groups in the LAT trial. (A) Rarefaction curves generated on the observed number of OTUs. Principal coordinate analysis (PCoA) of the bacterial communities in samples collected at 4 and 10 dph (B), 16 and 25 dph (C), 3 and 7 dpi (D) based on Bray–Curtis distances. Below and left boxplots show the overall distribution of PCoA 1 and PCoA 2 scores within each group and red asterisk (*) indicates significant difference compared to the control (NAB-S) group (* P < 0.05, ** P < 0.01, Wilcoxon rank-sum test). [file 40168_2023_1609_MOESM2_ESM.tif]

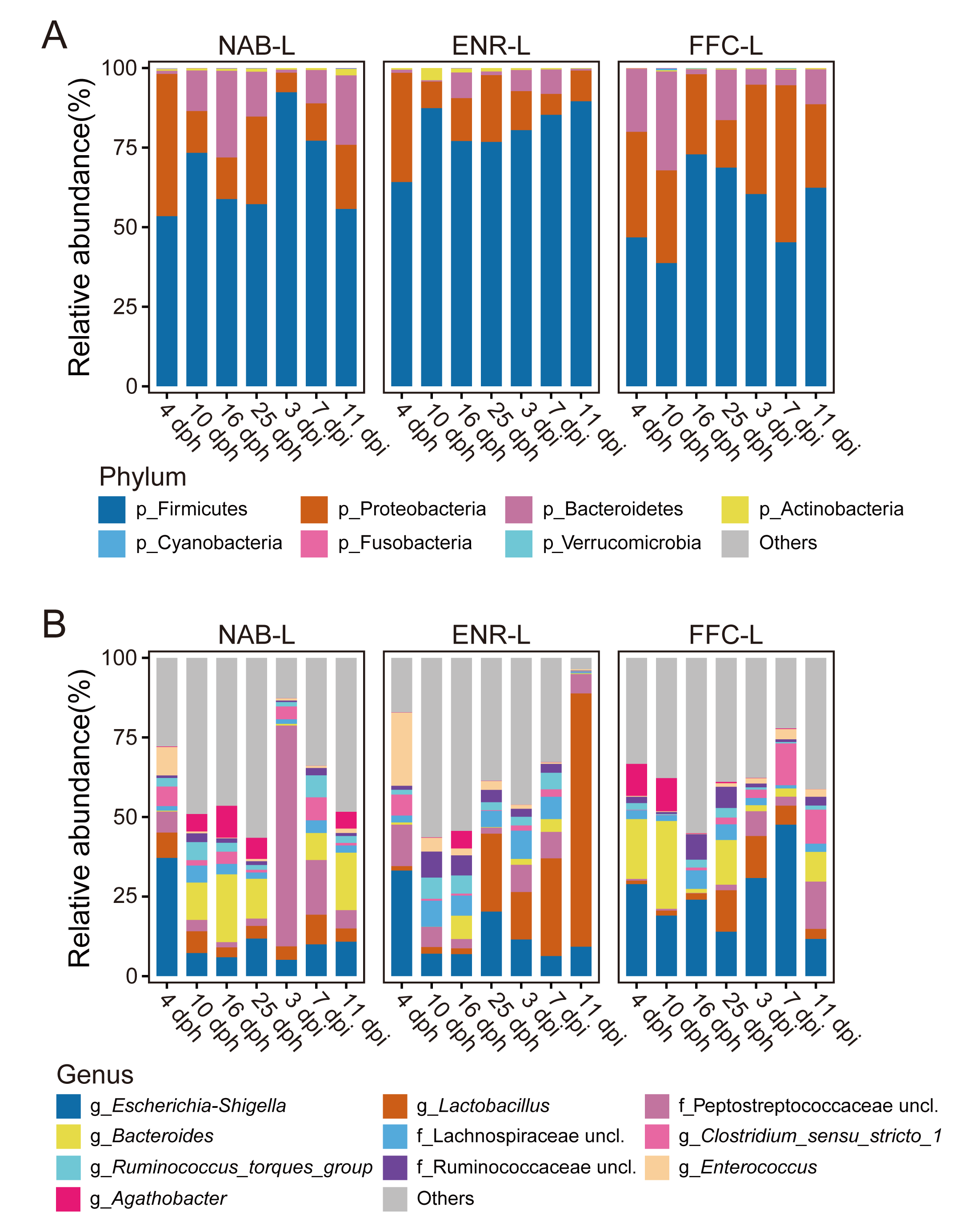

Supplement: Supplementary file 4 — Additional file 3: Supplementary Fig. 3. Composition and relative abundance of microbial communities in the different groups in the LAT trial. Stacked bar charts show phyla (A) and the top 10 most abundant bacterial genera (B). Each color represents the relative abundance of a bacterial taxon on the stacked bar chart. [file 40168_2023_1609_MOESM3_ESM.tif]

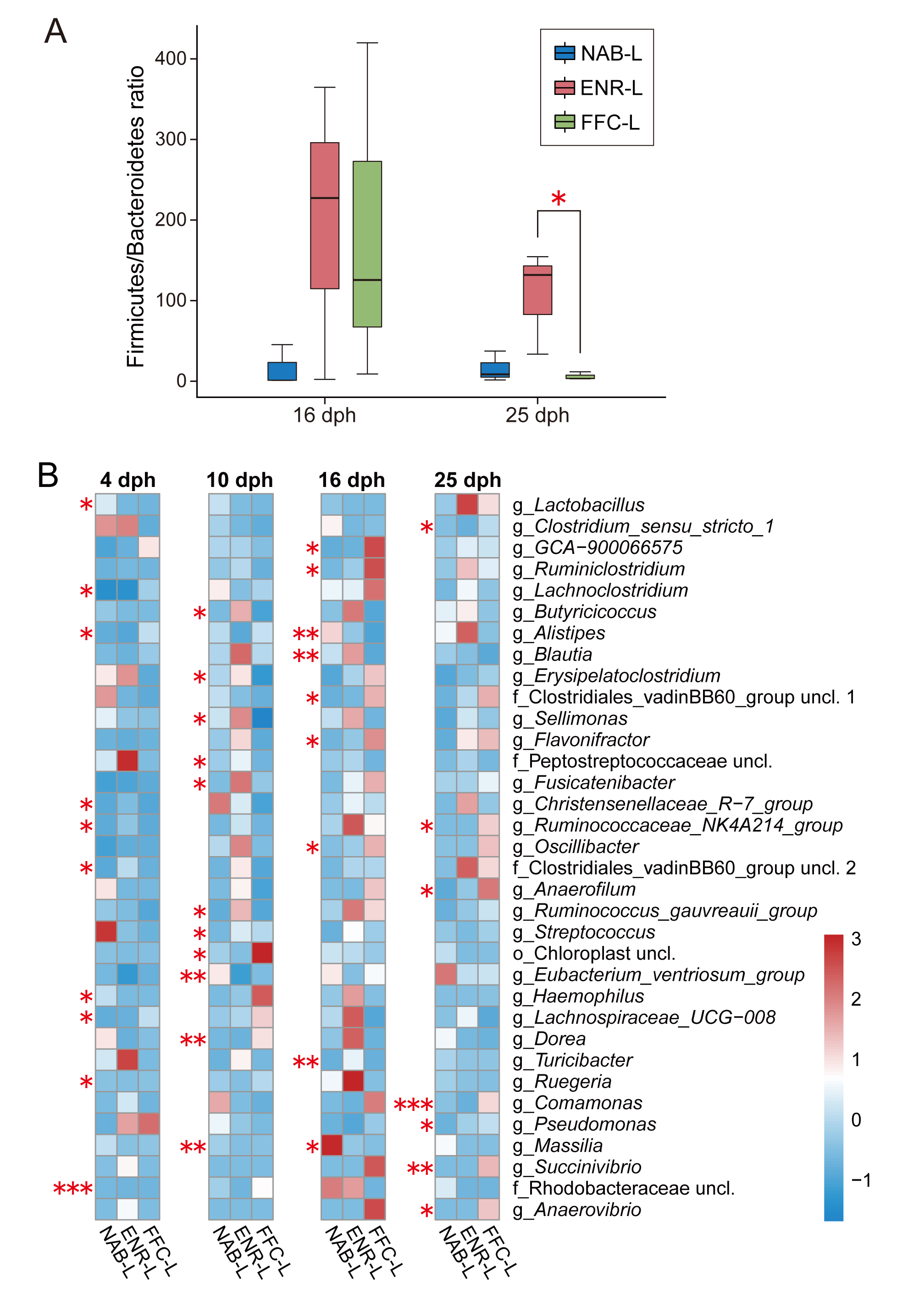

Supplement: Supplementary file 5 — Additional file 4: Supplementary Fig. 4. The differences of microbial communities among three groups in the LAT trial before H9N2 AIV infection. (A) Boxplots show the Firmicutes/Bacteroidetes ratio in the three groups at 16 and 25 dph. (B) Heat maps show the genera with significant differences between the three groups at 4, 10, 16 and 25 dph (* P < 0.05, ** P < 0.01, *** P < 0.001, ANOVA, Tukey HSD). [file 40168_2023_1609_MOESM4_ESM.tif]

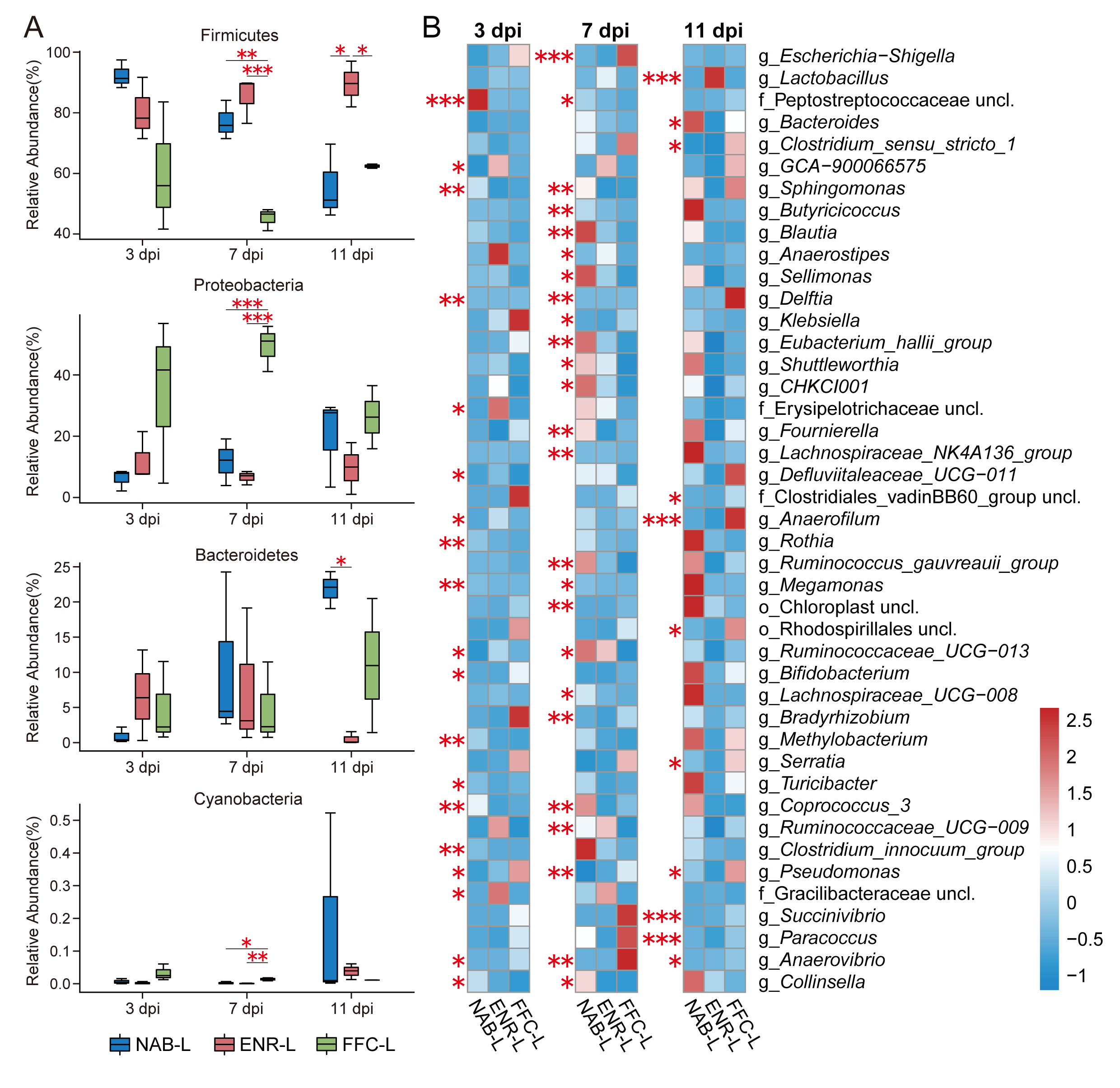

Supplement: Supplementary file 6 — Additional file 5: Supplementary Fig. 5. Differences in microbial communities among the three groups in the LAT trial after H9N2 AIV infection. (A) Boxplots showchanges in the four major phyla in the three groups at 3, 7 and 11 dpi. (B) Heat maps show the genera with significant differences between the three groups at 3, 7 and 11 dpi (* P < 0.05, ** P < 0.01, *** P < 0.001, ANOVA, Tukey HSD). [file 40168_2023_1609_MOESM5_ESM.tif]

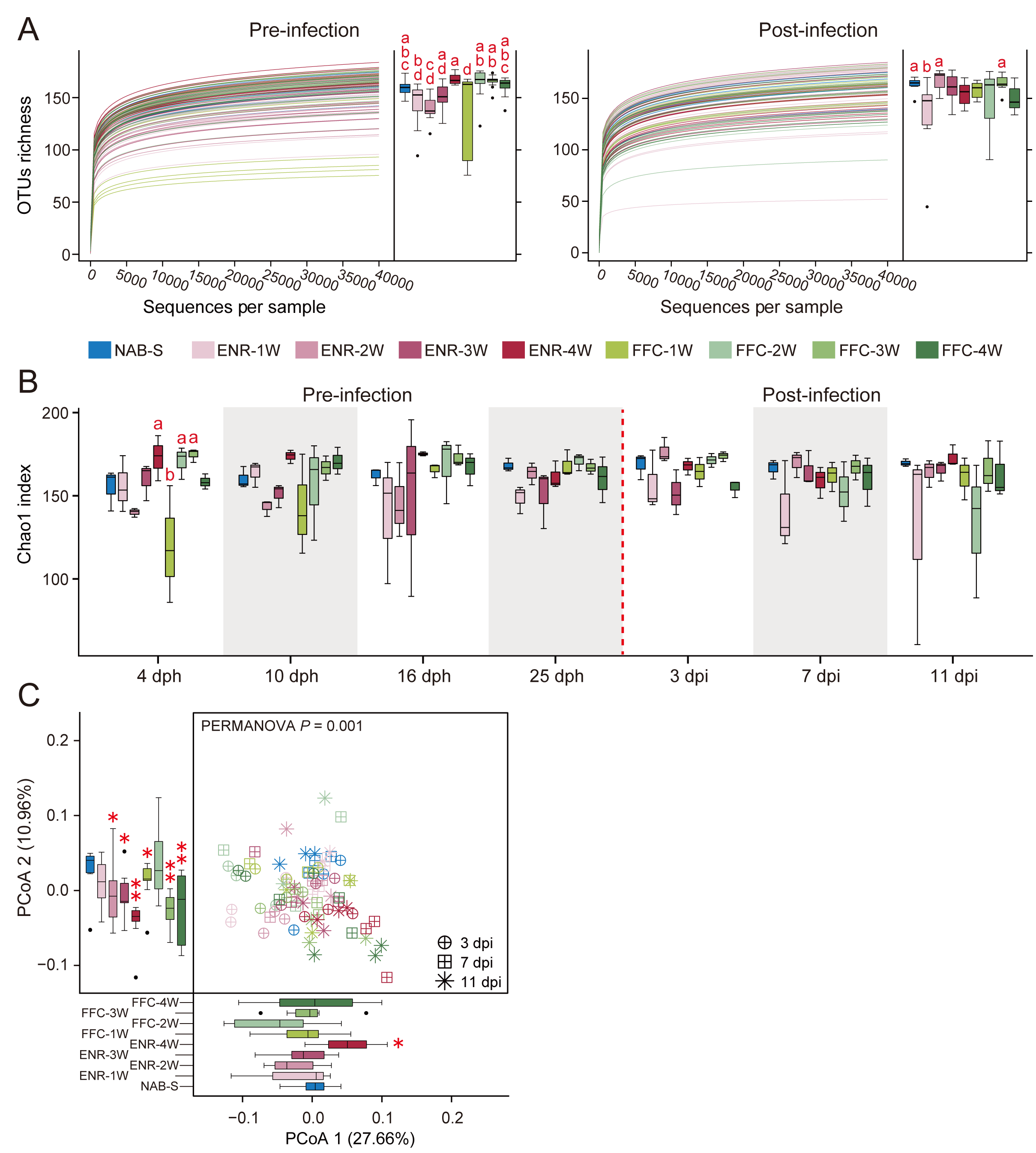

Supplement: Supplementary file 7 — Additional file 6: Supplementary Fig. 6. Shifts of gut microbial diversity in chickens that received short-term antibiotic treatment. (A) Rarefaction curves generated from observed numbers of OTUs. Boxplots on the right shows the overall distribution. (B) Boxplot shows the Chao 1 diversity index of gut microbiota in chickens with time. Different red lowercase letters denote statistical significance (P < 0.05, ANOVA, Tukey HSD test). (C) Principal coordinate analysis (PCoA) of the bacterial communities based on the Bray–Curtis distances for postinfection samples from the SAT trial. Two outliers from the ENR-1W and FFC-2W group at 11 dpi were removed from the plot. Below and left boxplots show the overall distribution of PCoA 1 and PCoA 2 scores within each groups and the red asterisk (*) indicates significant difference compared to the NAB-S group (* P < 0.05, ** P < 0.01, Wilcoxon rank-sum test). [file 40168_2023_1609_MOESM6_ESM.tif]

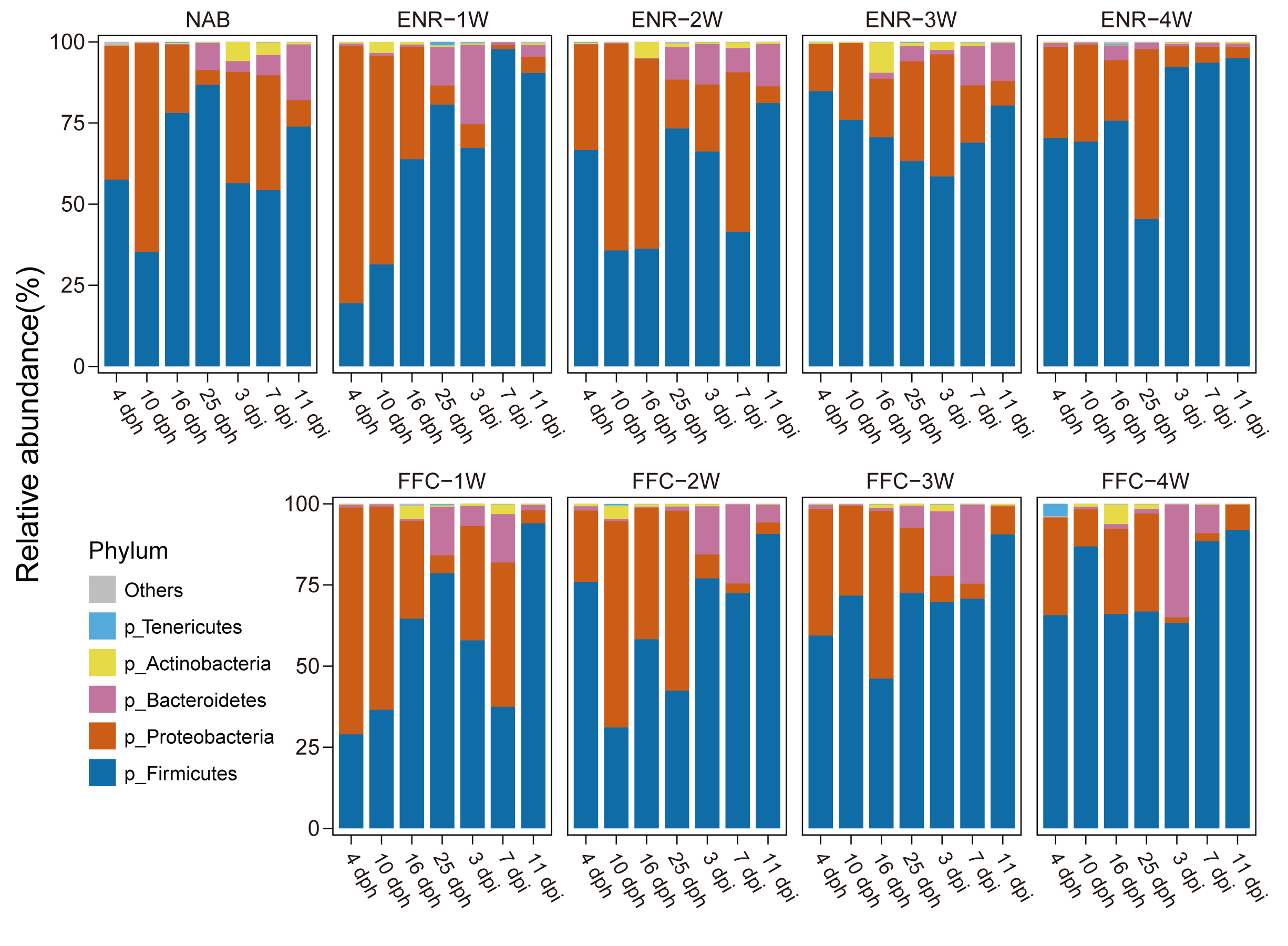

Supplement: Supplementary file 8 — Additional file 7: Supplementary Fig. 7. Relative abundance of the bacterial phyla in the different groups in the SAT trial. Stacked bar charts show taxa at the phylum level. Each color represents the relative abundance of a bacterial phylum on the stacked bar chart. [file 40168_2023_1609_MOESM7_ESM.tif]

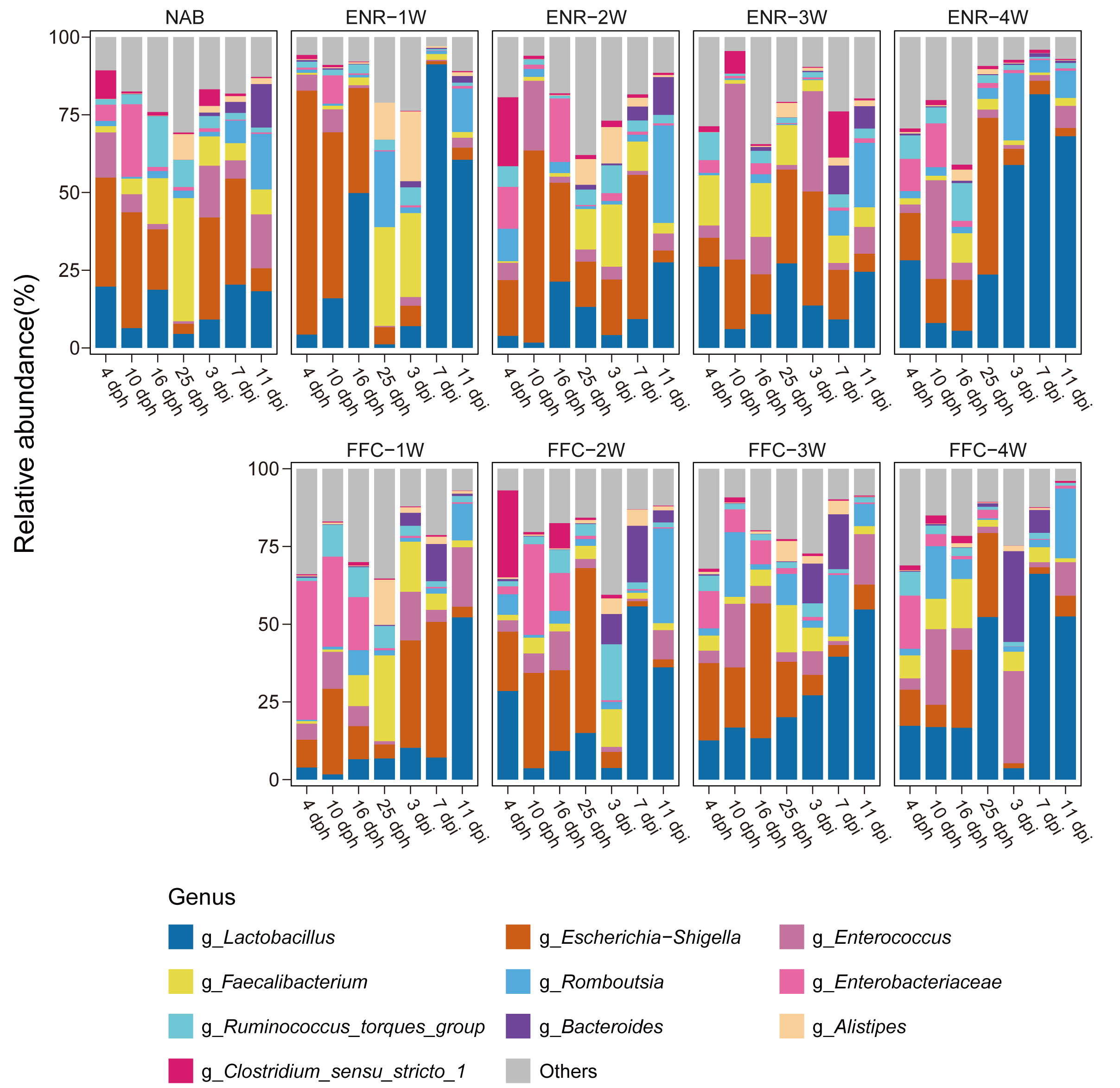

Supplement: Supplementary file 9 — Additional file 8: Supplementary Fig. 8. Relative abundance of the top 10 most abundant bacterial genera in the different groups in the SAT trial. Stacked bar charts show taxa at the genus level. Each color represents the relative abundance of a bacterial genus on the stacked bar chart. [file 40168_2023_1609_MOESM8_ESM.tif]

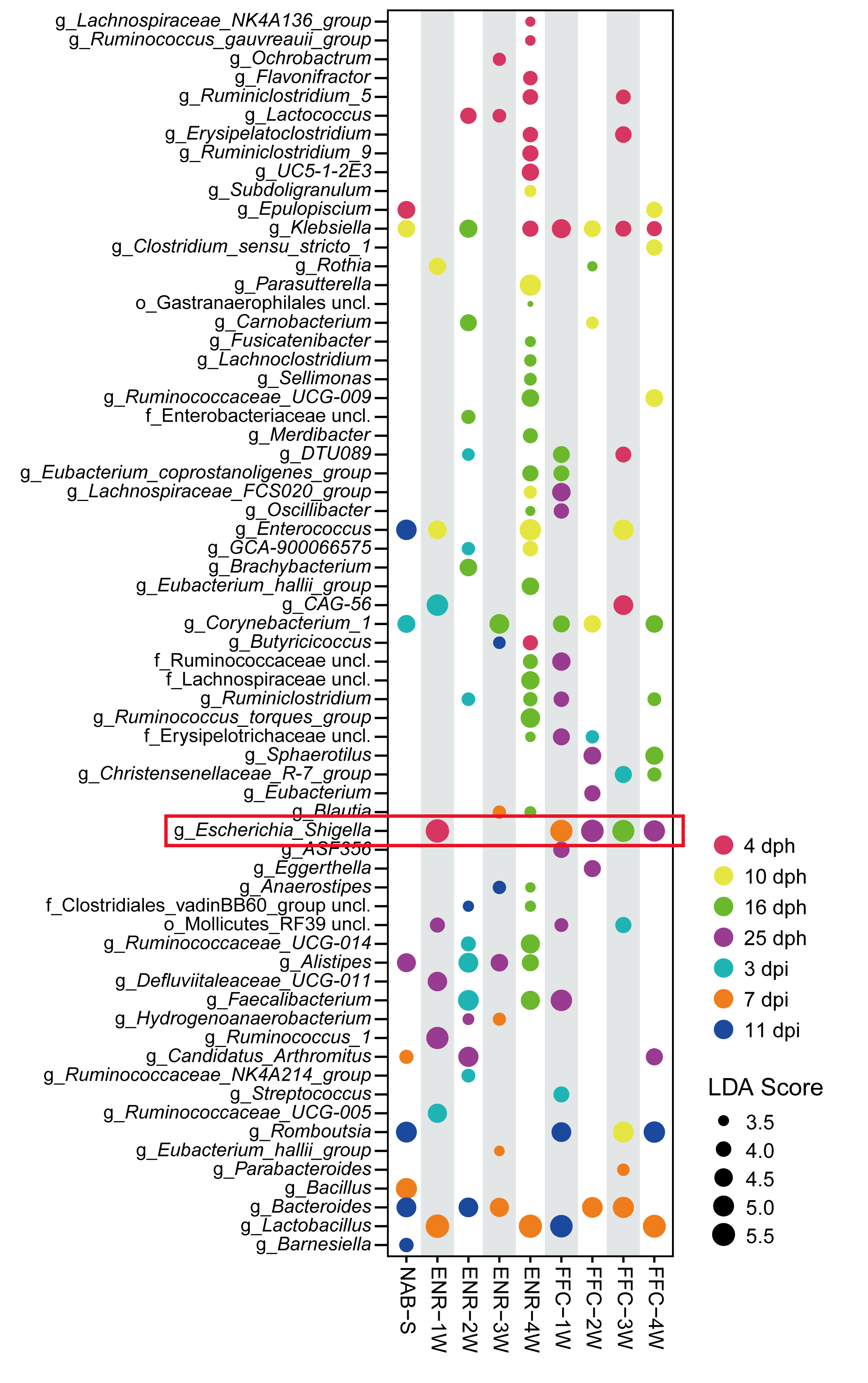

Supplement: Supplementary file 10 — Additional file 9: Supplementary Fig. 9. Strikingly different microbial genera of each group in the SAT trial. Dot plots show differentially abundant genera determined by LEfSe between the seven sampling time points in each group (P < 0.05, LDA score > 2). [file 40168_2023_1609_MOESM9_ESM.tif]

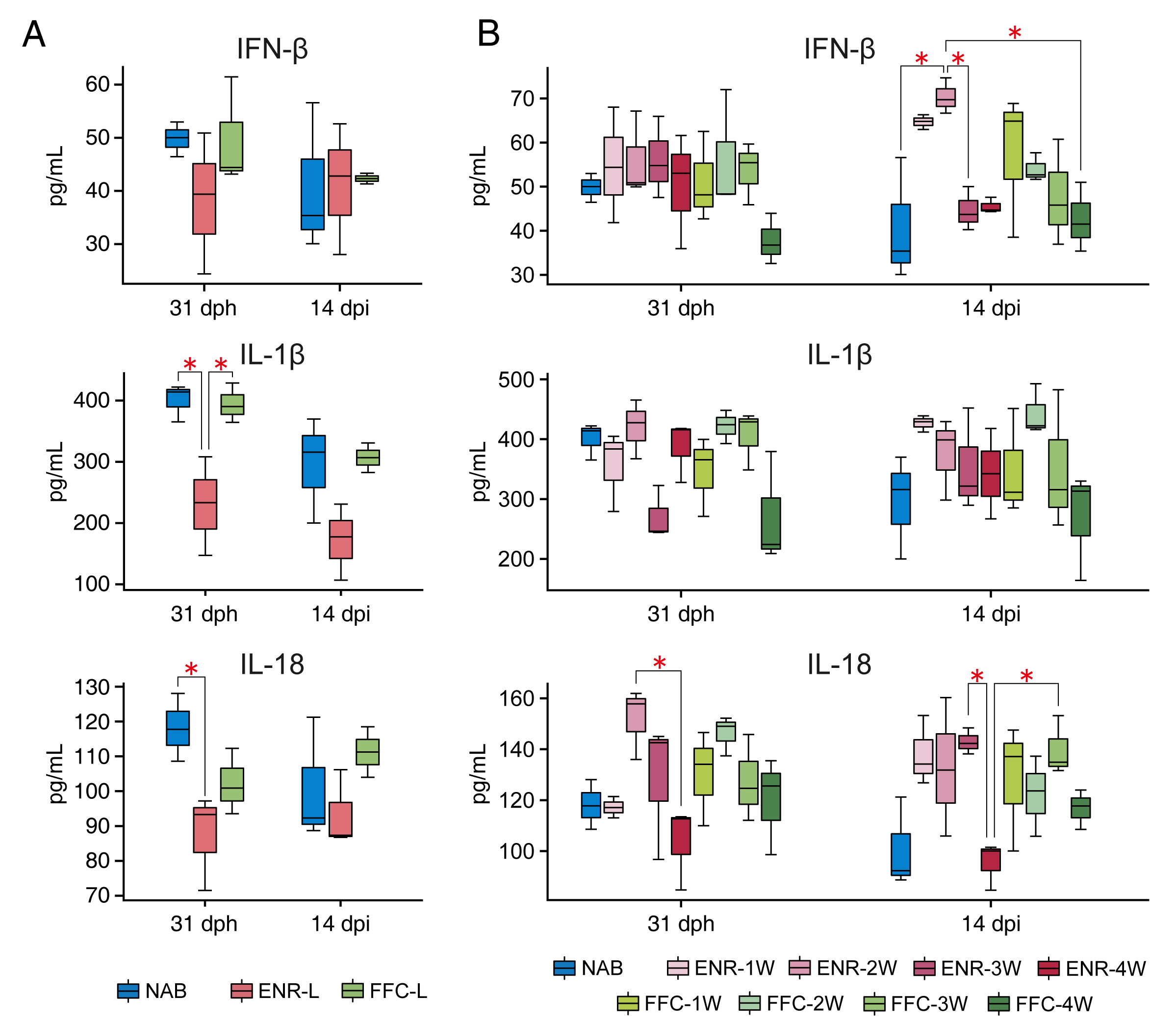

Supplement: Supplementary file 11 — Additional file 10: Supplementary Fig. 10. Levels of cytokines in the prophylactic antibiotic-treated chickens at 31dph and 14 dpi. Boxplot showing levels of IFN-β, IL-1β and IL-18 in serum collected from chickens in the LAT (A) and SAT trials (B) at 31dph and 14 dpi (* P < 0.05, Tukey HSD). [file 40168_2023_1609_MOESM10_ESM.tif]

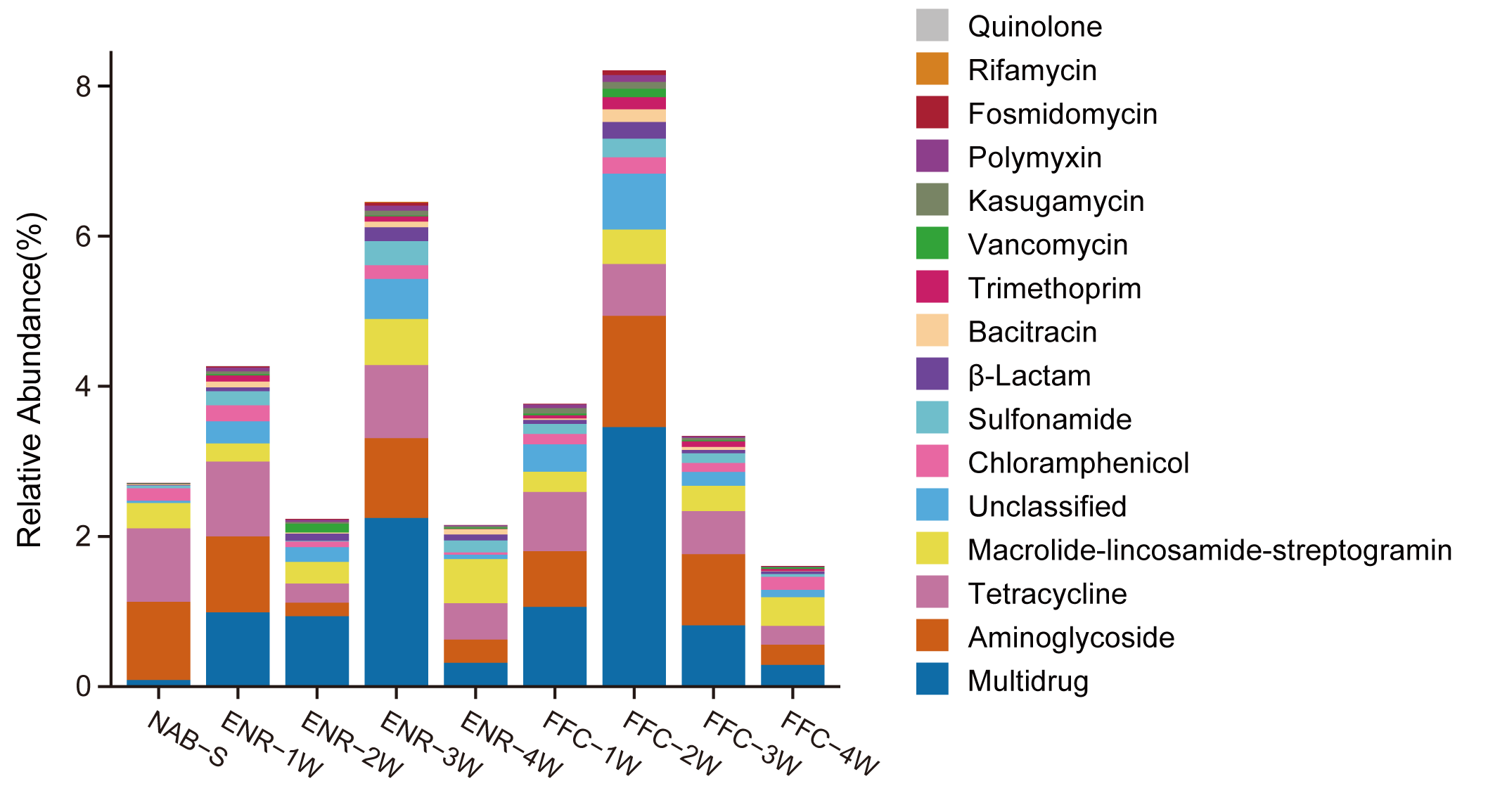

Supplement: Supplementary file 12 — Additional file 11: Supplementary Fig. 11. Relative abundance of ARGs in the different groups in the SAT trial. Stacked bar charts show relative abundance of ARGs. Each color represents the relative abundance of an ARG on the stacked bar chart. [file 40168_2023_1609_MOESM11_ESM.tif]
